# Moral judgements of fairness-related actions are flexibly updated to account for contextual information

**DOI:** 10.1038/s41598-020-74975-0

**Published:** 2020-10-20

**Authors:** Milan Andrejević, Daniel Feuerriegel, William Turner, Simon Laham, Stefan Bode

**Affiliations:** Melbourne School of Psychological Sciences, The University of Melbourne, Parkville, VIC 3010 Australia

**Keywords:** Social behaviour, Psychology

## Abstract

In everyday life we are constantly updating our moral judgements as we learn new information. However, this judgement updating process has not been systematically studied. We investigated how people update their moral judgements of fairness-related actions of others after receiving contextual information regarding the deservingness of the action recipient. Participants (*N* = 313) observed a virtual ‘Decision-maker’ share a portion of $10 with a virtual ‘Receiver’. Participants were aware that the Decision-maker made these choices knowing the Receiver’s previous offer to another person. Participants first made a context-absent judgement of the Decision-maker’s offer to the Receiver, and then a subsequent context-present judgement of the same offer after learning the Receiver’s previous offer. This sequence was repeated for varying dollar values of Decision-makers’ and Receivers’ offers. Patterns of judgements varied across individuals and were interpretable in relation to moral norms. Most participants flexibly switched from relying on context-independent norms (generosity, equality) to related, context-dependent norms (relative generosity, indirect reciprocity) as they integrated contextual information. Judgement of low offers varied across individuals, with a substantial minority of participants withholding their context-absent judgements of selfishness, and another minority that was lenient towards selfishness across both judgements. Our paradigm provides a novel framework for investigating how moral judgements evolve in real time as people learn more information about a given situation.

## Introduction

Staying principled in a world that is unfolding before our eyes requires us to update our moral judgements as we learn new information. Consider the following story (a modified version of the Heinz dilemma^1^):

Heinz robbed a pharmacy and stole some medicine. The pharmacist was a greedy man, who increased the price of a medicine after he found out that Heinz’s wife needed that medicine to survive. Heinz only saw one way to save his wife’s life, and that was to rob the pharmacy.

While most will agree that a robbery is an immoral act after reading the first sentence, many would change their mind about Heinz’s robbery after they learned that the motive behind it was to save a life. Moreover, as we find out that the pharmacist tried to profit from his misery, we may even begin to praise Heinz’s act as brave and morally virtuous. In this example, the context of the action changes the valence of the moral judgement. In everyday life, we are often required to judge actions as good or bad without knowing much about the context in which they occur. Additionally, we need to make judgements about situations as they unfold in real time. As we acquire new information in a serial manner, we need to reassess and update our moral judgements. However, how we do this has not been comprehensively studied. Research so far has mainly focused on isolated single-shot judgements, whereby an individual already has access to the relevant contextual information (for example see refs.^2,3^). Therefore, the prominent theories of moral judgement to this day^4–6^ implicitly assume a single-shot judgement, and do not explain how moral judgement occurs as information dynamically changes on a short time scale (several seconds). In the present study we investigated how people update their moral judgements in the face of new information and aimed to provide new insights into how our moral cognition flexibly adapts to account for context-dependent moral norms.

### Moral judgment updating

Moral judgements are influenced by various types of contextual information when that information is presented before a moral judgement is made. For example, moral judgements of interpersonal actions have been shown to depend on the relational status between moral actors and victims^7,8^, their social identities^9,10^, their economic class^11^, as well as the victim’s history of moral or immoral actions (i.e. their moral deservingness)^12^. Judgements of the morality of an action are also modulated by information about whether there were better choice alternatives available^13^, and whether there were some mitigating circumstances that make it difficult to avoid a moral violation^14^.

Although these studies have provided evidence that context modulates moral judgements, none of them presented information sequentially to change the context and investigate judgement updating in real time. If contextual information is presented after an initial decision is made, moral evaluators may become susceptible to several well studied effects including: the status quo effect—a tendency to maintain the current or previous decision^15^, the choice-induced preference effect—a tendency to increase preference for the chosen option^16,17^, the congeniality bias—a tendency to pay less attention to information that does not conform to an initially formed impression^18^, and anchoring—a tendency to make insufficient adjustments to starting estimates, or initial beliefs^19,20^. All of these biases work against changing initial judgements and integrating new contextual information. However, a recent study presented evidence that these effects play only a minor role when it comes to updating of moral judgements based on blame^21^. Participants in this study updated their initial blame judgements after receiving information regarding the violator’s intentions, reasons, and also information regarding the preventability of unintentional violations (i.e. contextual information presented within a narrative indicating the violator’s insight). The authors argued that this lack of susceptibility to biases may be due to vital importance of making accurate moral blame judgements, as inappropriate blaming of others can be socially costly. Whether moral judgements are also immune to these decision biases is still unknown.

Other investigations of moral judgement updating, however, have shown little to no change in initial judgements. One line of research investigated changes in moral judgement across dichotomous good/bad options, i.e. judgement reversals^22–24^. In these studies, no additional information was presented after the initial decision, and reversals were expected to occur as a result of an endogenous change-of-mind. Across these studies, reversals were hypothesised to be more frequent if the initial decision was deontological as opposed to consequentialist; however, no evidence was found that supported this hypothesis. Decision reversals were rare, occurring between 1.2 and 4.8% of all trials. Another study investigated moral judgement updating when participants were presented with reasons opposing the initial judgement^25^. The authors reported that people rarely changed their initial judgement after considering the provided reasons. Importantly, most of the reasons provided at this stage were appeals to moral rules emphasising and repeating aspects of the already presented scenario. The presented reasons did not add new information and did not change the context of the moral situation. In contrast to these studies, one study showed that people adjust moral judgements after learning information about the outcome of the judged action (i.e. how successful this action was)^26^. However, in this study participants reported their moral judgements by answering whether the judged action “should have been taken”, and not by specifically answering whether the action is morally good or not, which may have prompted practical as opposed to moral considerations when evaluating actions based on their outcome. Moreover, considering the general lack of adjustment in other studies it remains unclear whether and to what extent adjustments occur for moral judgements after new contextual information is provided.

Moral judgement updating can be studied in several ways. One of the most prominent approaches to study moral decision-making involves presenting participants with moral dilemmas. Moral dilemmas are typically presented as long paragraphs of text that are often susceptible to confounds (e.g., framing effects, a myriad of linguistic features), concern drastic and unlikely situations, and require elaborate consideration of many pieces of information^2,3^ (discussed in ref.^27^). Most of the moral judgements that we make in our daily lives, however, do not involve drastic situations, and do not require considering such a large number of factors^28^. Another approach to study moral decision-making is to present shorter sections of text, or ‘moral vignettes’^29^. This approach has been used in the abovementioned study investigating updating of blame-related judgements^21^, as well as others investigating effects of contextual information on moral judgements^7,8,30^. Although more ecologically valid, these stimuli are text-based and are subject to similar confounds. Moreover, this approach only allows the context to be varied in a discrete fashion (e.g., someone either did or did not have a good reason to do harm), but the contextual information we learn about in real-life is often more subtle. This means that, while these studies show that context matters, they do not illuminate how context-dependent moral norms shape judgement adjustments across a range of more subtle context manipulations. Yet another approach is to use economic game tasks as models of moral problems (especially concerning fairness) with tangible consequences^31,32^. Although these tasks are limited in the extent to which they can capture the full spectrum of real-world moral judgements, they are nevertheless well-controlled, have been shown to correlate with the real-world decisions^33^, and offer the possibility of parametrically modulating the moral stimulus^34^. Moreover, understanding of norms guiding distributive justice (i.e. fairness norms) has been of central importance to the study of morality^35^.

### Context-independent and context-dependent fairness norms

Fairness permits multiple interpretations—and frequently in previous research, multiple fairness norms have been identified across individuals doing the same task. When it comes to descriptive fairness norms, people generally tend to split resources into equal outcomes across individuals (equality norm^31,36,37^), but sometimes they also decide to distribute outcomes proportionally according to individual contributions to a shared resource (equity norm^38–40^). In distributive justice scenarios in which one actor decides to distribute resources to one or more others, additional norms may also play a role. These include a tendency to return previous favours (reciprocity norm^41,42^), or a tendency to assign more to others than to oneself (generosity norm^43,44^). If an actor needs to share with others who have a history of moral or immoral actions, they show a tendency to share proportionally to others’ past moral behaviour (indirect reciprocity norm^45,46^), or otherwise scale the outcomes to their perceived moral deservingness^47^.

Within this list of fairness norms we can distinguish two kinds of norms: *context-independent norms*, such as equality and generosity, which are principles that can be applied to any distributive situation independent of contextual information regarding past behaviour, and *context-dependent norms*, such as equity, reciprocity and indirect reciprocity, which are contingent on the contextual information regarding past individual contributions to the shared resource, or past moral behaviours in general. Most previous research has studied fairness by giving participants tasks in which they can choose to behave in line with either context-independent or context-dependent norms, exposing that they find one more important than the other^48–50^. For instance, previous research has shown that individual preferences for equity vs. equality relate to people’s economic interests^51^, and their political stance^52^. Studies investigating single distributive decisions, reflecting either context-independent norms or context-dependent norms, do not allow for investigation of relationships between context-independent and context-dependent norms. In the present study we investigated how decisions in the absence of context relate to subsequent decisions made after contextual information is revealed, allowing us to investigate the relationship between the two decisions and their underlying norms.

Although the relations between context-independent and context-dependent norms have not previously been assessed, several expectations can be derived from previous research on the relative importance of those norms for morality judgements in general. For instance, when third-party observers are well-informed about the relative contributions of each party to the common good, and in absence of any personal interest, a context-dependent equity norm is used in a judgement more often than the context-independent equality norm^48^. Similarly, context-independent equality is used more often than context-dependent reciprocity only in exceptional situations, in which reciprocity generates extreme outcome disparities^49^. However, which fairness norms people rely on, and consider to be more important, appears to be malleable and susceptible to change within minutes if the circumstances are altered. For example, people will switch between relying on equity and equality as they learn which of the two incurs most economic benefits to themselves^51^. We thus expect that, although there may be some primacy of context-dependent norms, people in most instances flexibly switch from adhering to context-independent to adhering to context-dependent norms as they learn about relevant context.

There is another complicating factor in many of the abovementioned studies that makes it difficult to cleanly delineate context-dependent and context-independent norms. Much of the fairness research presented above investigated behavioural adherence to norms—i.e. whether participants’ behaviour is consistent with a norm. However, adherence to the kinds of norms described above requires sacrifice of self-interest. In order to better understand fairness norms without considerations of personal costs or gains to the participant, moral decision-making can be studied in situations in which the participant is a third party and unaffected by the outcomes of the observed actions^30,53^. Moreover, fairness norms can be studied not only by investigating decisions in distributive justice tasks^54^, but also by investigating third-party evaluations of distributive actions. Third-party evaluation tasks, in which participants report their beliefs about what other people may find socially appropriate, have been used to identify socially shared norms^55^. In such tasks, third-party observers can also report their personal moral judgements across a broad range of distributive actions. In the absence of other confounds, patterns of moral endorsements and condemnations can be interpreted as evaluative normative profiles (i.e. patterns of judgements based on distinct moral norms).

### The present study

**Figure 1:**
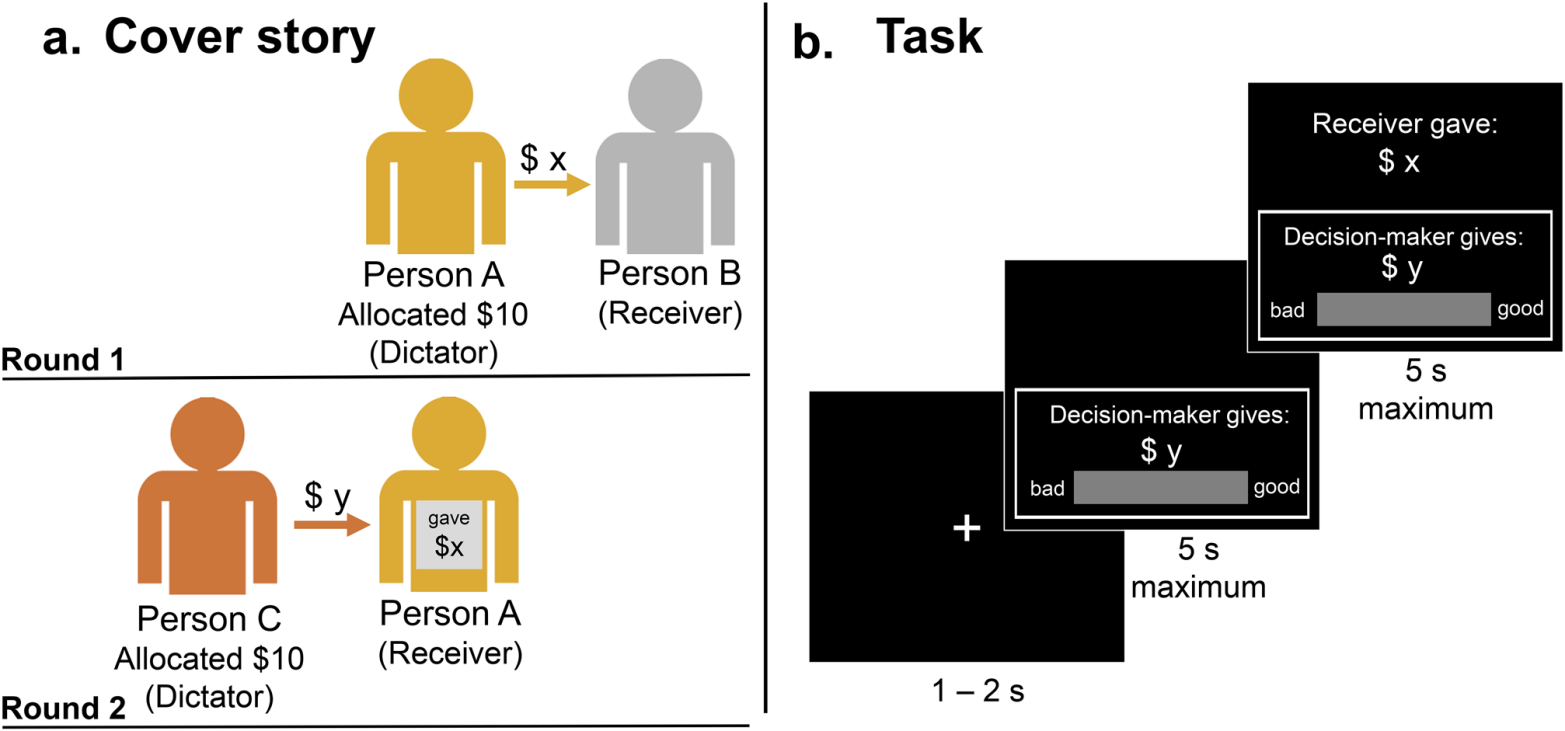
Experimental paradigm. (**a**) Depiction of the cover story presented to participants before completing the experiment. In Round 1, Person A played the role of the Decision-maker, was given $10, and decided how much of that amount they would give to their partner, Person B. In Round 2, Person A became the new Receiver. A new person, Person C, became the Decision-maker, was given $10, and decided how much they would give to Person A. Importantly, Person C could see how much Person A gave in the previous round (*$x*). Person C decided to give a certain amount (*$y*) to Person A. Person C is the only player whose moral actions (sharing behaviour) is judged in the real experiment. (**b**) Trial sequence. Participants were presented with the amount that the Decision-maker (i.e., Person C) gave to the Receiver (i.e., Person A) in Round 2 without revealing the context for this decision (amount *$x*). Participants used their mouse to indicate their judgement of the Decision-maker’s action on a scale ranging from morally “good” to “bad”. After this, the context was revealed (i.e., the amount that the Receiver had given in the previous round; *$x*). Participants again indicated their judgement of the Decision-maker’s action in a new judgement.

In this study, we examined how normative judgement profiles in the absence of contextual information relate to context-present normative judgement profiles as participants update their judgements in real time, narrowly focusing on moral judgements of distributive justice. We asked participants to make moral judgements regarding sharing behaviour in situations in which actors share a monetary resource with a single partner, i.e. a variant of the Dictator Game^56^ (see Fig. 1). In this game a “Dictator”, a virtual person referred to as the “Decision-maker” throughout this paper, is given $10 and decides how much of this amount to share with another person termed the “Receiver”. Participants in our study made two subsequent judgements about the Decision-maker’s action in relation to how morally “good” or “bad” they perceived the action to be on a continuous scale. To do this, they first observed the Decision-maker’s action and made an initial, context-absent judgement. Following this, they were presented with information regarding how much the Receiver had previously given to another person (when acting as the Decision-maker towards a different Receiver in the same game, which crucially was observed by the current Decision-maker). Participants then made a new judgement (i.e. a context-present judgement) of the same Decision-maker’s action. Across the duration of the experiment participants made moral judgements across the full range of possible values of the Decision-maker’s and the Receiver’s sharing amounts and combinations of such. We expected several norms to be relevant for people’s context-absent judgements of different ranges of Decision-maker offers in this task. Medium offers (~ $5) indicate that Decision-makers evenly split their allocated money, and participants may interpret this as an instance of morally good behaviour based on the equality norm. High offers (> $6) indicate that the Decision-maker gave more than they kept for themselves, and participants may interpret this as behaviour that is in accordance with the generosity norm. Low offers (< $4) indicate that the Decision-maker acted in accordance with their own economic interest, and may be interpreted as selfish behaviour. Similarly, we expected several context-dependent norms to be relevant for different ranges of Decision-makers’ offers relative to Receivers’ offers. If two offers are similar, this indicates that the Decision-maker treated the Receiver the way the Receiver treated another person, and participants may interpret this as an instance of morally good behaviour according to the indirect reciprocity norm. Offers of Decision-makers that are higher than those made by Receivers may instead be interpreted as relative generosity. Offers of Decision-makers that are lower than those made by Receivers indicate that the Decision-maker failed to indirectly reciprocate the Receiver’s offer, and may be interpreted as relative selfishness.

We investigated two main research questions: (1) as outlined above, decision-making is susceptible to multiple biases which could potentially limit moral judgement updating in real time. We therefore investigated whether presentation of relevant context would lead to adjustment of initial judgements. (2) Assuming adjustments of initial judgements were observed, we were also interested in whether this updating process occurs in a systematic fashion. In other words, do people switch between the norms they use as they learn contextual information? Context-independent norms and context-dependent norms may shape judgement profiles across two judgements. If individuals find context-independent norms more important than context-dependent norms, they may make judgements before knowing contextual information and may not adjust their judgements after exposure to contextual information. To explain, individuals who strongly endorse equality may judge an even split of 10 dollars between the Decision-maker and the Receiver as morally good across both judgements, regardless of what they learn about the Receiver’s previous action. Individuals who strongly endorse generosity may judge high offers as morally good, and equal offers as more neutral, regardless of what they learn about the Receiver’s previous actions. Similarly, individuals who strongly condemn selfishness may judge low offers as morally bad across the two judgements. If, on the other hand, individuals find context-dependent norms more important, they may withhold making strong initial judgements until the contextual information is available. For example, an individual relying on indirect reciprocity norms may, in the absence of context, judge high, even, and low offers as neutral, and only decide to endorse or condemn them once they learn whether these match the past sharing behaviour of the Receiver. Lastly, if individuals flexibly rely on multiple norms depending on what kind of information is available, they may rely on context-independent norms to make their initial judgement and update their judgement flexibly to reflect context-dependent norms after context is presented. For instance, individuals, who flexibly rely on equality and indirect-reciprocity norms may initially endorse even splits between the Decision-maker and the Receiver and condemn low (and/or high offers), may subsequently adjust to endorse low/even/high offers that are proportional to Receivers past offers, while condemning those that are not. Others, who flexibly rely on generosity and relative generosity, may initially endorse high and condemn low offers, and subsequently adjust to condemn high offers if they are lower than previous offer made by the Receiver, or endorse low offers if they are still higher than the past offers of the Receiver.

We characterised the patterns of judgements across sharing amounts using functional principle components analysis (fPCA) to investigate whether participants flexibly updated their judgements to account for presented contextual information, and to test how variation across individuals in the context-absent judgements related to variation in judgement styles when contextual information was available (see “Methods” section for details). Principle component scores from this analysis method reflected the degree to which participants’ patterns of decisions were influenced by different context-absent and context-present fairness norms. We hypothesised that participants would rely on context-independent moral norms (equality and generosity) when making their context-absent judgements and rely on context dependent-norms (indirect reciprocity) when making context-present judgements. We also hypothesised that within-participant judgment patterns reflecting the importance people assign to particular norms in context-absent judgements (e.g., generosity) would positively correlate with patterns reflecting the importance of similar context-dependent norms in context-present judgements (e.g., relative generosity).

## Results

### Context-absent judgements

**Figure 2:**
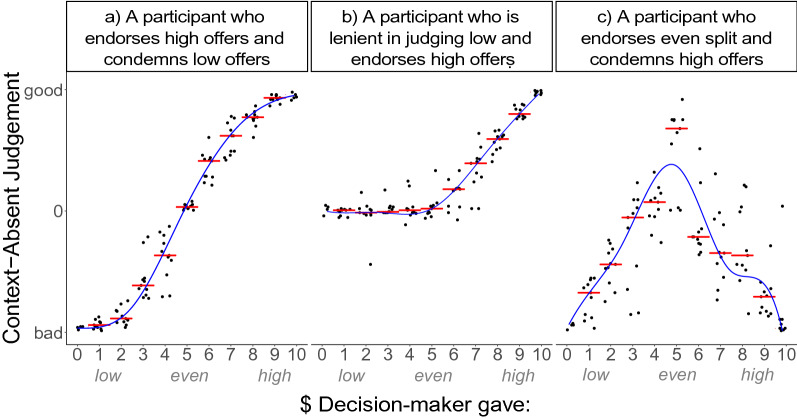
Context-absent judgement. Examples of three participants with different judgement styles. Judgement on each trial is plotted against the Decision-maker’s offer. Red bars represent the median judgement for the given offer. The blue line represents a smoothed functional data analysis fit over the median judgement data.

To characterise context-absent (CA) moral judgements and identify moral norms latent in those patterns, we used fPCA. Examples of functional fits for the three most prominent patterns in the data can be seen in Fig. 2. Inspection of the scree plot revealed a three-component solution, which accounted for 93.8% of the variability in the data. These three components reflect dimensions of variability in normative judgement profiles. The first CA principle component (CA-PC1), accounting for 44.8% of variability in the data, captured individual variability in judgements of Decision-makers who gave over $6 (Fig. 5a). Individuals with higher CA-PC1 scores judged high offers by the Decision-maker ($7–$10) as more morally good. To note, most participants judged these high offers as good on average, and only a small minority (4.47%) judged them as morally bad. The second component (CA-PC2), accounting for 39.5% of variability, indicated the level of lenience in moral condemnation of Decision-makers who gave less than $4 (Fig. 5b). Individuals with higher CA-PC2 scores judged Decision-makers who gave little ($1–$3) as less morally bad. Most participants judged these low offers as bad, however a small minority (5.43%) placed their judgements only slightly above the middle of the scale on average. The third component (CA-PC3), accounting for 9.5% of the variability, reflected judgements of Decision-makers who gave between $3 and $7 (Fig. 5c). Individuals with higher CA-PC3 scores judged Decision-makers who gave an even-split (or close to even-split) amount as more morally good. Most participants judged the middle offers as neither good nor bad responding to the middle of the scale on average. However, a minority of 16.29% of participants, judged middle offers as morally good, with their average judgements leaning more than 15% from the scale centre towards the “morally good” end. As a comparison, only 7.02% of participants rated 5$ offers as morally better than 10$ offers.

These three principle components divide the variability in judgements of Decision-maker offers into three parts—judging high, low, and even offers. These can be interpreted as reflecting three different context-independent norms, namely generosity, selfishness and equality, respectively. Participants appeared to vary in how strongly they endorsed and condemned Decision-makers’ actions in line with these norms in their context-absent judgements (see the judgement profiles by PCA scores depicted in Fig. 5a–c). To explain, CA-PC2 appears to reflect general lenience in condemning selfishness, and higher scores on this component reflected more positive ratings when the Decision Maker gave $4 or less (Fig. 5b). CA-PC1 and CA-PC3 appear to reflect the general tendency to endorse generosity and equality, respectively. Given that participants did not yet know the context of the Decision-maker’s action when making the context-absent judgement, these components can also be interpreted in relation to how strongly they reflect withholding of extreme judgements (i.e. importance people assign to context-dependent norms in context-absent judgements). In particular, CA-PC2 may reflect withholding condemnation of seemingly selfish Decision-makers in the absence of contextual information. Similarly, other participants appear to vary in how strongly they endorsed high (CA-PC1) and even (CA-PC2) offer by the Decision-maker before they find out the relevant contextual information. We have adopted these interpretations for further analyses below.

### Context-present judgements

**Figure 3:**
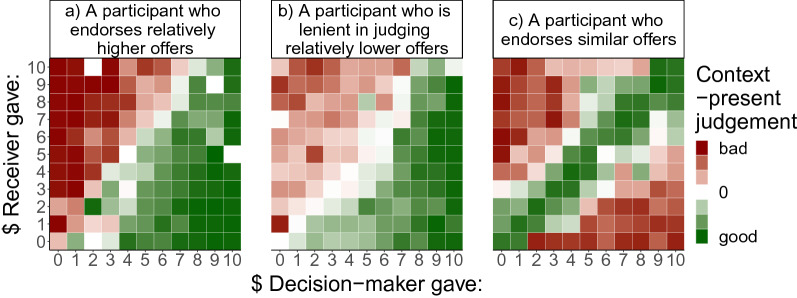
Context-present judgement examples. The judgement is represented by the colour of the tiles arranged in the Decision-maker’s action and context combination space. Separate plots are shown for three participants with different judgement styles, highlighting examples of (**a**) endorsing relative generosity, (**b**) lenience in judging relatively low offers, and (**c**) endorsement of indirectly reciprocal offers.

**Figure 4:**
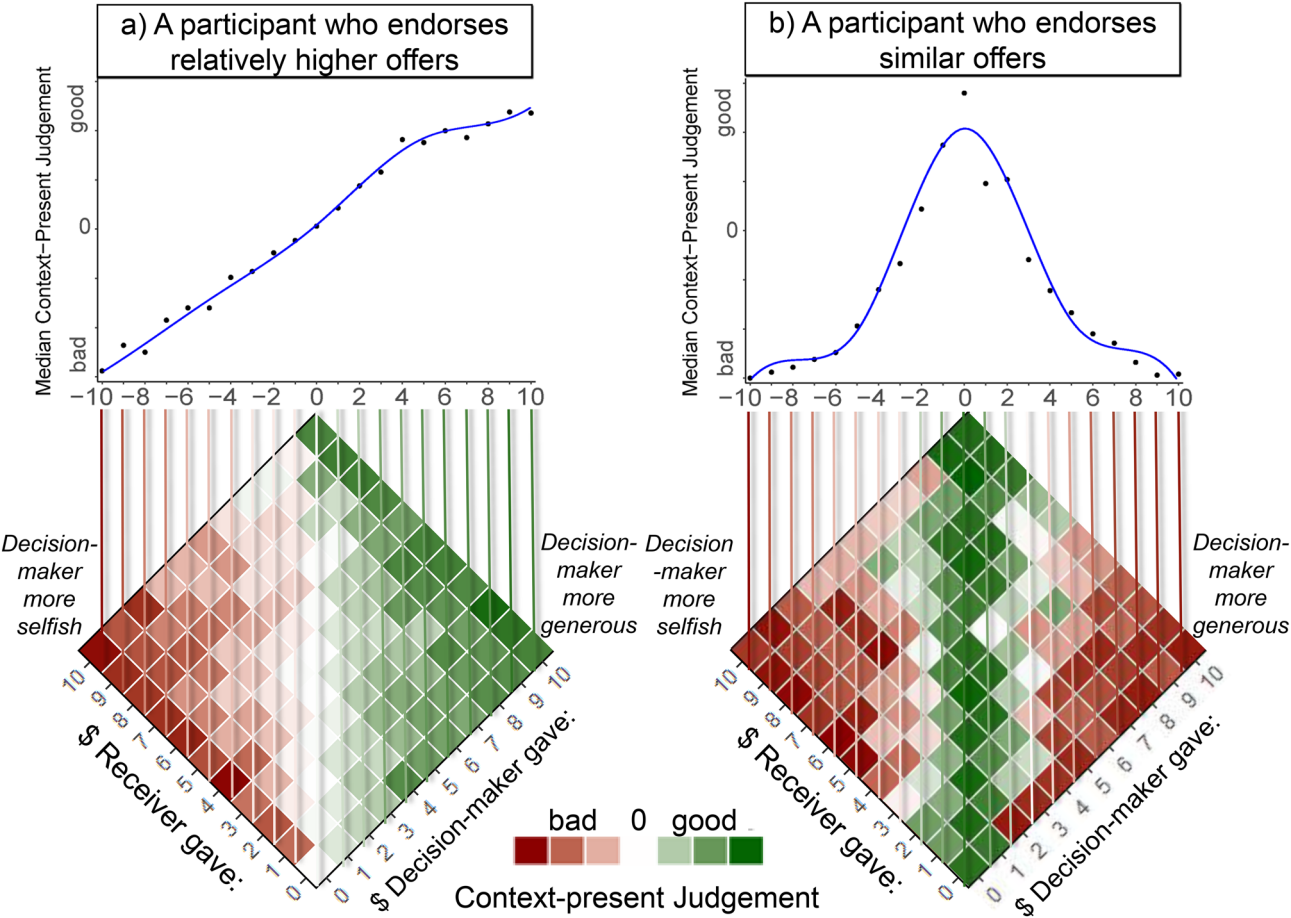
Context-present Judgements for a range of Decision-makers’ offers relative to Receivers’ (− 10 to 10). Plotted in (**a**,**b**) are examples of two participants. Each point on the scatter plot represents the median judgement value over the corresponding groups of tiles in the tile-plot (also see Fig. 3). This correspondence is designated using vertical coloured lines. The blue line in the scatter plot represents a smooth functional data analysis fit over the median judgement data.

**Figure 5:**
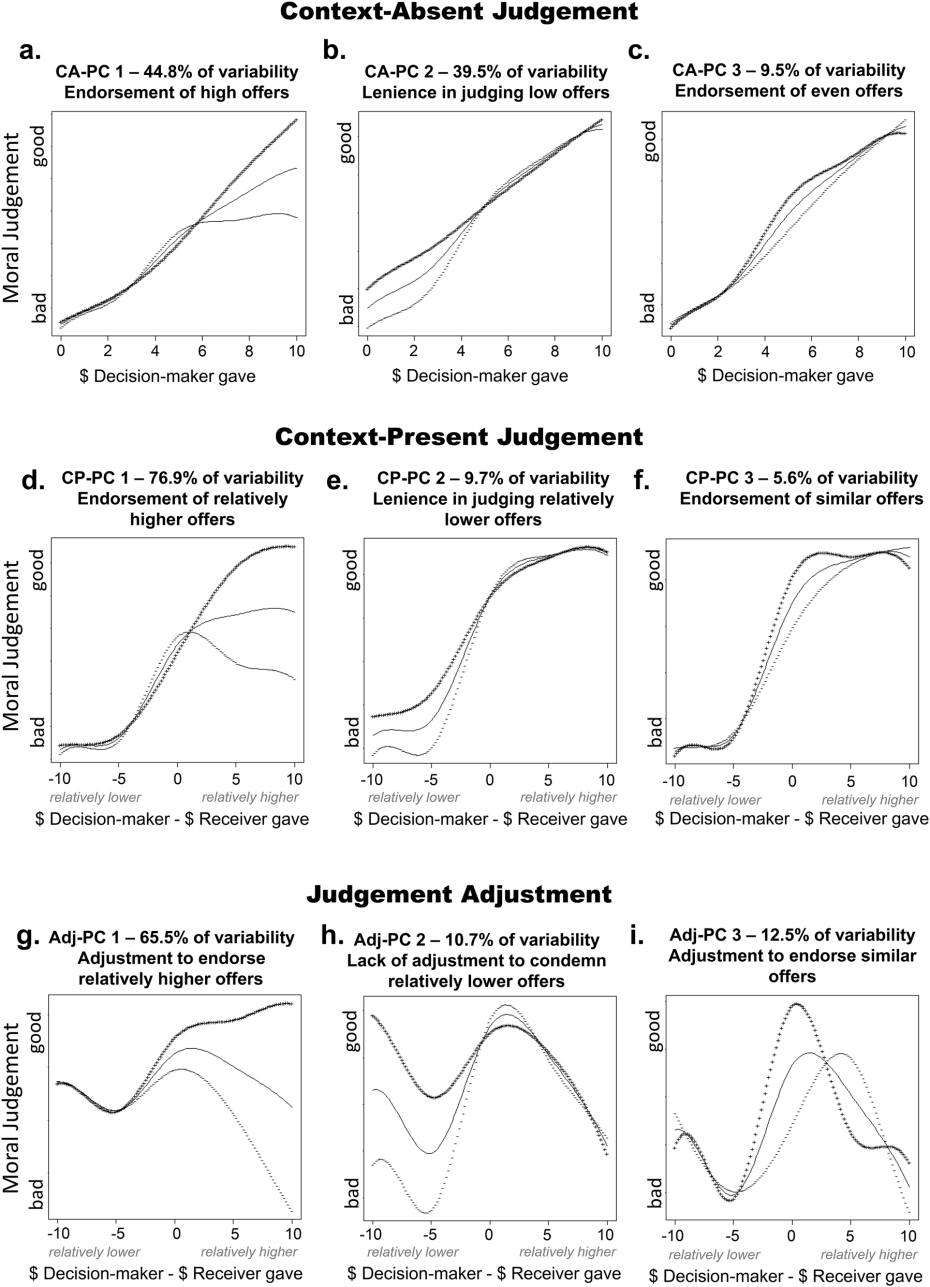
Individual differences in judgement profiles. Principle Components (PC) for context-absent judgements (**a**–**c**), context-present judgements (**d**–**f**), and judgment adjustment (context-present judgement—context-absent judgement) response patterns (**g**–**i**). Mean judgement curves for two judgements are plotted as a continuous line. Curvilinear variability in the data along each principle component is plotted using + and − signs. These two curves are calculated by adding and subtracting 2 standard deviations of each of the principle components to the mean. Values for which these curves diverge denote trial types in which participants’ responses differed according their scores on each principal component.

To characterise context-present moral judgements and identify moral norms latent in these judgements, fPCA was applied to the judgement profiles. Examples of three most prominent patterns in the data can be seen in Fig. 3. Examples of functional fits over the judgement data across a range of Decision-makers’ offers relative to Receivers’ offers can be seen in Fig. 4. The scree plot revealed a three-component solution which accounted for 91.2% of variability in the data. The first component (CP-PC1), accounting for 75.9% of variability in the data, captured inter-individual variability in judgements of the Decision-makers’ offers that were larger than those of the Receivers. Individuals with high CP-PC1 scores endorsed Decision-makers who gave upwards of $2 more than their Receiver partner did in the previous round (Fig. 5d). Most of our participants endorsed these offers, while a minority of 18.21% responded below the scale centre, judging these offers as morally bad. The second component (CP-PC2), accounting for 9.7% of variability across participants, indicated the level of lenience in moral condemnation of Decision-makers’ offers that were smaller than Receivers’ (Fig. 5e). Individuals with high CP-PC2 scores were more lenient in judging Decision-makers’ offers upwards of $3 less than their Receiver partners gave in the previous round. To note, almost all participants qualified these offers as bad, and only a small minority (0.63%) responded just above the scale centre on average. The third component (CP-PC3), accounting for 5.6% of variability, indicated individual variability in judgements of Decision-makers’ offers that were similar to Receivers’ (Fig. 5f). Participants with high CP-PC3 scores endorsed Decision-makers who had given the same amount to the one Receivers gave in the previous round. Most of our participants endorsed these offers, with 77.95% responding above the scale centre on average. Notably, however, 32.06% of participants judged similar offers as morally better than higher offers on average.

These three principle components divided the variability in judgements of Decision-maker offers, considered relative to the Receiver’s previous offers, into three parts—relatively higher, relatively smaller, and similar offers. These parts can be interpreted as reflecting three context-dependent norms, namely relative generosity, relative selfishness and indirect reciprocity. As shown in Fig. 5d–f participants varied in how strongly they endorsed and condemned the Decision-maker’s contextualised actions in line with these norms. To explain, CP-PC1 and CP-PC3 appear to reflect the general tendency to morally endorse relative generosity and indirect reciprocity, respectively, while CP-PC2 appears to reflect general lenience in judging relative selfishness.

### Judgement adjustments

On average, participants adjusted their responses after receiving contextual information by 16.72% of the scale length in either direction (*SD* = 7.28, 95% CI [15.92, 17.53]). Participants changed the valence of their judgement in an average of 22.08% of completed trials (*SD* = 15.28, 95% CI [20.66, 23.49]).

Functional PCA was also used to analyse individual variability in judgement adjustment patterns (Adj; i.e. the extent to which participants changed their moral judgements after receiving relevant contextual information). The resulting three component fPCA solution accounted for 88.7% of the variability in the data. The first component (Adj-PC1), accounting for 65.5% of variability in the data, captured individual variability in adjustments (ranging from negative to positive) upon learning that the Decision-maker gave more than the Receiver (Fig. 5g). High Adj-PC1 scores reflected stronger positive adjustments to endorse Decision-makers after learning that they gave more than Receivers had given in the previous round. The second component (Adj-PC2), accounting for 10.7% of the variability, related to adjustments upon finding out the Decision-maker gave less than Receiver (Fig. 5h). High Adj-PC2 scores reflect the lack of change in participants’ moral judgements compared to the preceding context-absent judgement, and low scores reflect stronger negative adjustments. The third component, explaining 12.5% of the variability (Adj-PC3), reflected individual variability in the degree of adjustments upon finding out that Decision-makers’ offer was similar to Receivers’ previous offers (Fig. 5i). Participants with high Adj-PC2 scores adjusted their responses to be more positive upon finding out that the Decision-maker gave the same amount as the Receiver had given in the previous round.

Similar to those components identified in analyses of context-present judgements, the principle components identified here divide the variability in judgement adjustments into three parts, corresponding to three context-dependent norms: relative generosity, relative selfishness, and indirect reciprocity. The component scores can thus be interpreted as measures of importance people assign to these context-dependent norms. However, this interpretation has an important caveat. In instances in which the initial evaluation strongly relied on context-independent norms, and participants made a response close to the limits of the response scale, there was no option for further adjustment in the same direction. In other words, the extent of adjustments that could be made in a particular direction was somewhat dependent on the initial response, and potentially limited for extreme judgements. For example, if a participant maximally endorsed generosity in their context-absent response, they could not give an even more positive response to relative generosity, due to the bounded limits of the scale. Therefore, stronger expressions of these components might (also) be related to participants’ subjective importance of context-dependent norms over context-independent norms, whereby relevant contextual information is anticipated by withholding extreme positive or negative judgements, leaving room for subsequent adjustment once the context is presented.

### Relations between context-absent and context-present judgement patterns

After characterising the dominant patterns of moral judgements in terms of moral norms, and the importance that people assign to each when making moral judgements, we investigated how the importance of norms correlated across the two judgements. To do so, we correlated the scores of principle components obtained from the first and second judgements. As our principle component scores were not normally distributed, non-parametric Spearman’s rank correlation coefficients were calculated. Overall, there were strong correlations between importance of context-independent norms and their relative context-dependent counterparts. CA-PC1 was positively correlated with CP-PC1 (*r*_*s*_ = 0.461, *p* < 0.001), indicating that those who endorsed high offers in context-absent judgements also endorsed relatively high offers in context-present judgements. CA-PC2 was positively correlated with CP-PC2 (*r*_*s*_ = 0.557, *p* < 0.001), indicating that those who were lenient in judging low offers in context-absent judgements were also lenient in condemning relatively low offers in context-present judgements. CA-PC3 was positively correlated with CP-PC3 (*r*_*s*_ = 0.470, *p* < 0.001), indicating that those who endorsed even split in context-absent judgements also endorsed matching offers in context-present judgements. These correlations between importance of context-independent norms and their relative context-dependent counterparts were stronger than correlations between context-independent norms and other context-dependent norms (e.g., CA-PC1 and CP-PC1 correlation was higher than that between CA-PC1 and CP-PC2, and that between CA-PC1 and CP-PC3). A full matrix of correlations between judgement patterns is included in the Supplementary Table S1.

### Relations between context-absent patterns and updating patterns

Above we discussed two possible explanations for the lack of either condemnation or endorsement in a pattern of judgements: that it may reflect weaker general tendencies to endorse or condemn behaviours that fall within a domain of a particular context-independent norm, and that it may reflect withholding of extreme judgements in anticipation of contextual information. The latter account would predict larger decision adjustments upon receiving contextual information. We tested this by correlating context-absent patterns with patterns of adjustment. CA-PC1 did not correlate with Adj-PC1 (*r*_*s*_ =  − 0.078, *p* = 0.167), which did not support the hypothesis that CA-PC1 reflected withholding of judgements (i.e. assigning more importance to context-dependent than to context-independent norms). CA-PC2 was strongly negatively correlated with Adj-PC2 (*r*_*s*_ =  − 0.610, *p* < 0.001), indicating that those who were lenient in judging low offers in their context-absent judgment, had stronger negative adjustments upon learning that the Decision-maker’s offers were relatively lower than those previously made by the Receiver. This result is in line with the notion of withholding strong judgements in the context-free phase in anticipation of (potentially negative) contextual information. CA-PC3 was weakly positively correlated with Adj-PC3 (*r*_*s*_ = 0.143, *p* = 0.01), indicating that those who endorsed even splits in context-absent judgements, had stronger adjustments to endorse Decision-maker’s offers when they were similar to those previously made by the Receiver.

As many participants judged high offers as very good in their context-absent judgements, and so did not have an option to further adjust their judgement positively, this raises a concern that random variation in context-present judgements may contribute to false detection of negative adjustments. To explain, if a participant makes a judgement that is maximally ‘Good’ by clicking on the end of the scale for their context-absent judgement, then they cannot adjust their judgement to be even better once they have learned the relevant contextual information in that trial. They can, however, adjust their decision toward ‘Bad’ in this trial. Additionally, any imprecision in the mouse cursor position can only result in judgement adjustments toward the ‘Bad’ direction of the scale in this example. This dependence means that random variation (i.e. noise) in the context-present judgement data will be more likely in the direction towards the middle of scale. Such noise would mask positive relationships between context-absent judgements that reflect tendencies to make extreme judgements and principle components that reflect adjustment of these extreme judgements. To account for this, we ran additional analyses to assess to what extent the absence of evidence for a positive relationship between CA-PC1 and Adj-PC1 could be explained by this dependency. If the relationship was masked by this dependency in the data, we would expect to unmask a positive relationship if we exclude participants who made extremely high context-absent judgements (i.e. those who had very high CA-PC1 scores). We performed these correlation analyses excluding 20% and 40% of participants with highest CA-PC1 scores (i.e. participants that had very little or no room to adjust positively), and we still found no evidence for a positive relationship between CA-PC1 and Adj-PC1 (for *n* = 252, *r*_*s*_ = 0.036, *p* = 0.569; for *n* = 189, *r*_*s*_ = 0.006, *p* = 0.932). Similarly, many participants judged low offers as very bad, and did not have an option to adjust their judgement negatively. In this case, noise could hypothetically contribute to false detection of positive adjustments of extremely low judgements of low offers, and boost the negative relationship between CA-PC2 and Adj-PC2. To address this issue we repeated the same analyses excluding 20% and 40% of participants with lowest CA-PC2 scores (i.e. participants that had very little space to adjust), and found that the negative effect observed between CA-PC2 and Adj-PC2 remained strong (for *n* = 252, *r*_*s*_ =  − 0.51, *p* < 0.001; for *n* = 189, *r*_*s*_ =  − 0.52, *p* < 0.001).

### Additional post-hoc regression analyses

In the sections above we report that those who were lenient in judging low offers in context-absent judgements were consistently lenient in condemning relatively low offers in context-present judgements (i.e. a positive relationship between CA-PC2 and CP-PC2). Conversely, we also report that those lenient in judging low offers in their context-absent judgment, also had stronger negative adjustments once they found out that the offer was relatively low (i.e. a negative relationship between CA-PC2 and Adj-PC2). One potential explanation for these opposite effects is that they coexist independently in our sample due to two different trajectories of progression of two judgements that participants took: (1) some of those that were lenient in condemning low offers in context-absent judgement were also overall lenient in condemning relatively low offers; (2) others who were lenient in condemning context-absent judgements, adjusted more negatively to condemn relatively low offers. To test this idea, we used a multiple linear regression analysis to predict CA-PC2 based on CP-PC2 and Adj-PC2. This regression model was statistically significant *F*(2,310) = 1299, *p* < 0.001, with an *R*^2^ = 0.893. There were significant effects of both CP-PC2 (*β* = 0.736, *SE* = 0.017, *t* = 39.07, *p* < 0.001), and CP-Adj2 (*β* =  − 0.738, *SE* = 0.019, *t* = 39.15, *p* < 0.001), providing evidence that indeed both judgement tendencies co-existed in our sample.

## Discussion

We developed a novel task in which participants made moral judgements of others’ actions. In this task participants observed the outcomes of a Dictator game, in which a Decision-maker shared a proportion of 10 dollars with a Receiver. Participants judged different Decision-makers’ offers in absence of any context and made subsequent judgements after they were presented with contextual information related to this action, i.e. the Receivers’ past offers when acting as a Decision-maker in a previous round of the game. We found that people adjusted their moral judgements after learning contextual information (by 16.62% of the scale length on average across all trials), and they did so in a systematic way. Participants flexibly transitioned from relying on context-independent norms (e.g., generosity, equality) to relying on context-dependent norms (e.g., relative generosity, indirect reciprocity) after learning this contextual information. Our findings form the basis of a novel framework for understanding how people update their moral judgements of sharing behaviour as they learn new contextual information.

### Judgement profiles before and after learning relevant contextual information

Participants were remarkably consistent in their judgement patterns across the testing session, yet there were stark differences in judgement styles among participants. The principle components derived from these judgement patterns captured the bulk of the inter-individual variability, allowing us to identify judgement styles that were influenced by several distinct fairness-related norms. For judgements made before contextual information had been presented, three separate components characterised how individuals judged low (< $4), approximately even-split, and high (> $6) offers by the Decision-maker. These three components can be seen as an organizing framework for people’s moral preferences in simple distributive justice problems and can be interpreted as the extent to which participants’ judgements relied on selfishness, equality and generosity norms. Critically, our design and analysis strategy allowed us to estimate the relative importance of these norms for each individual. A majority of participants found the generosity norm important and judged high offers as good. However, a smaller portion of participants found equality to be more important than generosity. These individual differences are compatible with previous research showing that multiple norms can be used to distribute resources, and in particular that both generosity and equality norms influence people to act against their economic interest and give up money^38,40,44,50^, as well as with previous research showing that norms influence people’s moral decisions when their economic interest is not at stake^30,53^. Our findings extend this to moral judgements, showing that in situations where economic interest is not at stake, generosity and equality norms have varying degrees of influence across individuals in their third-party moral judgements. We also found that in addition to generosity and equality, selfishness permits multiple interpretations. Previous research examining sharing behaviour has not discussed norms for selfishness as separate from equality and generosity, and sharing low portions has been interpreted as behaviour motivated by self-interest^44,50^. In our task, participants’ judgements posed no monetary cost to them, in contrast to the first-person dictator game, yet still some participants were more lenient in judging low offers than others, and this variability was orthogonal to variability associated with high and medium offers. This indicates that, in addition to norms endorsing generosity and equality, norms condemning selfishness might also shape individuals’ preferences for distributive justice. These norms condemning selfishness may impact people’s sharing behaviours and judgements independently from self-interest, and future studies should explore this possibility by correlating the importance of selfishness norms with measures of sharing behaviour.

For judgements made after learning relevant contextual information, we identified three components which corresponded to the influence of context-dependent norms. Individuals showed substantial variation along principle components corresponding to judgements of relatively higher, relatively lower, and similar offers relative to what the Receiver had previously given. These principle components can be seen as an organizing framework for people’s moral preferences in distributive justice problems in which information about the deservingness of each individual is salient. These principle components can be interpreted as reflecting the importance of three context-dependent norms (relative generosity, relative selfishness and indirect reciprocity). Our results suggest that, while most participants relied on each of these norms, the relative importance of each norm was not equally distributed and varied across individuals. Relative generosity was more important than indirect reciprocity to most people, but a notable portion of people had the opposite preferences (32%). Some of these people also condemned relatively higher offers, which may reflect the importance that people assign to retribution (e.g., that someone who behaved poorly in the past should not receive generosity but should be punished). Our principle component findings are in line with previous research showing that people account for deservingness of moral actors when sharing resources by either reciprocating^45–47^, or exceeding the past favour of their partner^57,58^. Moreover, our findings that a portion of people found rewarding selfish actions to be morally inappropriate, are in line with previous research showing strong individual differences in punishment of selfishness^42,59,60^. Variation in punishment across individuals, with only small number of punishers has been shown to be beneficial for reinforcing moral standards and sustaining cooperation within larger groups^61,62^.

### Adjustment of judgements after learning contextual information

Importantly, our results not only show that people use context-dependent norms in making moral judgements, but that they adjust their judgements substantially after context was presented, and in 22% of the trials even crossed over from one side of the scale to another, indicating a change from “good” to “bad”, or the other way around. This finding is in line with previous studies showing that contextual information modulates moral judgements^8,12^, as well as findings by Monroe and Malle that moral blame judgements are updated after presentation of reasons containing contextual information^21^. While Monroe and Malle theorised that judgement updating should happen in blame judgements specifically, as people are highly motivated to assess blame correctly, our results suggest that updating also occurs in moral judgements relating to fairness norms. There might, however, be other differences between more general moral judgements and blame judgements, for example in adjustment size, which we did not explicitly assess here. Our findings starkly contrast with other previous research studying updating that reports rare and small judgement adjustments^22–25^. For instance, we found changes in moral judgement valence to occur in 22.3% of the trials on average, whereas these studies reported 1.2–4.8%. These previous studies, however, have investigated either endogenous changes-of-mind or adjustments after presentation of reasons persuading to change mind, without introducing new contextual information. Higher rates of changes in decision valence in our study is likely due to presentation of relevant contextual information. Additionally, the continuous judgement scale used in our study may be more sensitive to adjustments as compared to binary choice sets used in previous studies. Our findings highlight that moral judgements are highly susceptible to changes when made in dynamic situations, and are subtly adjusted in response to incoming information. However, for an adjustment to happen, it may be essential that this information pertains to moral norms. Importantly, these findings are not compatible with previous suggestions that status quo effects, decision induced preference, congeniality effects, as well as anchoring and adjustment substantially diminish real-time updating of moral judgement.

### Transitions from context-independent to context-dependent norms

Our results show that participants who endorsed high offers in context-absent judgements also endorsed relatively high offers in context-present judgements. Similarly, those who endorsed even-split offers in context-absent judgements also endorsed similar offers in context-present judgements, and adjusted more strongly to endorse similar offers. These results are in line with our hypothesis that people flexibly switch from relying on context-independent norms (generosity, selfishness, equality) to relying on related context-dependent norms (relative generosity, relative selfishness, indirect reciprocity) depending on the availability of the context, in order to maintain coherence in judgements with an overarching moral principle, or virtue. This finding supports the idea that the set of norms on which people rely is susceptible to change depending on the availability of contextual information. This finding is also in agreement with previous research showing that fairness norms change within minutes if circumstances are altered^51^. Overall, the norms that exert the greatest influence over moral judgements appear to be highly dependent on information available, and that to understand moral norms of various contextual contingencies, we need to study how contextual information modulates which norms we place importance on, adding one piece of information at a time. Nuanced manipulations of contextual information could be highly valuable for advancing our understanding of other context-dependent norms both in distributive justice (e.g., equity, reciprocity) as well as other moral domains (e.g., revenge, defence).

Our findings outline systematic relationships between the importance of specific context-independent norms and specific context-dependent norms to individuals. While much of the existing literature conceptualises context-dependent and context-independent norms as opposites^48,49^, the strong relations in our study contrarily may reflect general virtues or values. For example, the observed relationship between the endorsement of equality and endorsement of indirect reciprocity may reflect a general tendency to endorse balanced (reciprocal and equal) distributions of resources. Endorsement of generosity and endorsement of relative generosity may reflect general moral endorsement of the generosity virtue. These coherent relationships within virtues were stronger than relationships between unpaired norms. This interpretation introduces the idea of a latent order (i.e. a taxonomy) of context-dependent and context-independent moral norms. So far moral psychology has systematically studied individual differences in the importance people assign to broader domains (e.g., moral foundations), with fairness being only one domain compared with harm, purity, loyalty, and authority^63^. Within the fairness domain, there have been studies showing strong differences in sharing behaviour across individuals^48–50^. However, individual differences in fairness virtues have not been studied systematically. One possible interpretation of our results is that more abstract concepts such as a generosity virtue, or a symmetry virtue (equality, indirect reciprocity) guide our moral cognition of sharing, and that appropriate norms are used in situations where contextual information is or is not available. Future research may investigate whether the importance people assign to virtues in judgement of simple sharing scenarios generalise to other and more complex distributional justice situations in experiments as well outside the lab.

### Individual preferences for context-dependent norms

Participants’ responses to low offers (< $4) showed a different updating pattern to that described in the previous section. Our results suggest that there were two updating trajectories that participants took: (1) some of those that were lenient in condemning selfishness in context-absent judgement were also lenient in condemning relatively low offers, which may reflect the underlying context-invariant virtue for condemning selfishness, and (2) others who were lenient in condemning context-absent low offers adjusted more negatively to condemn relatively low offers after learning the context. These findings support the view that some participants withheld their judgements when faced with a low offer and adjusted more strongly once they learned the context, indicating that these individuals found context-dependent norms (relative-selfishness) more important than context-independent norms (selfishness) even before the context was revealed. These findings are in line with previous research showing that people rely on context-dependent norms when additional contextual information exists^48^. However, since withholding of judgements primarily occurred in trials with low offers, this raises a question why low offers are special in this regard. One possibility is that this is related to the negative valence of these judgements. Speculatively, judgements that someone’s action is “morally bad” may require more evidence and a higher degree of certainty, as condemnation can be socially costly (as is the case for blame judgements), and can damage relationships^21,64^. Because it is important for people to avoid incorrect or premature blaming, people may adopt context-dependent norms immediately in their judgement of low offers when they are aware that contextual information exists. When it comes to judgements of high offers, mistaking actions as morally good may not be as important, and context-independent norms favouring positive judgement can be adopted with less concern for context that is still unavailable.

### Limitations and future directions

Our findings regarding relationships between initial judgement styles and the extent of judgement adjustments should be interpreted with caution, as the level of noise in the data (e.g., due to imprecisions in mouse clicking) could potentially boost or mask these relationships. We explained this potential issue in the “Results” section. However, we also provided evidence that the observed relationships were not strongly affected by this noise by demonstrating that correlation coefficients remained very similar when we excluded participants for which we suspected this noise to have the highest influence on ratings. Future studies may attempt to resolve this issue by altering the design and incorporating a measure of participants’ desire to alter their previous judgement positively or negatively after contextual information is presented (for example see ref.^65^). This would give participants the opportunity to signal the importance of contextual factors in trials where they had initially made judgements at the extreme ends of the good/bad spectrum. Another limitation of this study was that some participants might have had suspicions around whether they are judging actions of real people. Although we found that this was not the case in a small piloting sample, there is a chance that some people in our much larger study sample had doubts. Participants’ trust of the cover story is a very important factor for typical economic games paradigms in which people make decisions with economic implications. However, this might be less important for the study of third-party moral judgements, as morality researchers typically present imaginary scenarios to people^28^. Moreover, any judgement patterns that may hypothetically result from a realisation that the cover story is not true (e.g., random or invariant responding, change in strategy), were included as exclusion criteria in this study. Future studies could closely investigate potential differences between third party moral judgements of real and imagined scenarios. Another limitation relating to generalisability of our findings is that, while making initial judgements, our participants had expectations that they were going to learn contextual information, but moral judgement updating in everyday life may often occur without such expectancy. This expectancy may modulate both initial judgements as well as contextual updates. For example, this expectancy may make individuals perceive the information available as incomplete, initial judgements as imperfect, and result in more cautious decision-making. Additionally, this expectancy may prepare participants to adjust their judgements, inducing demand effects. Ideally, moral judgement updating should also be studied in scenarios in which information changes are not expected; however, this is difficult to study in multi-trial tasks, as repeated introduction of contextual information across trials inevitably gives rise to expectations. Future studies could also consider manipulating these expectancies parametrically in blocked or between-subject designs. Another limitation of this study was that we could not reliably measure response times, due to multiple sources of noise associated with responding using a computer mouse. Response times may offer additional insight into the temporal dynamics of the moral judgement process and improve our understanding of the processes involved in judgement updating (e.g., anticipation and integration of contextual information). Future studies may use more controlled mouse-click designs or button presses to measure response times. Finally, since our study methods were not publicly preregistered before the experiment was conducted, it would be beneficial for future research to replicate our findings using alternative methods and stimuli.

Our findings suggest that people substantially adjust their judgements in information-dynamic situations, which is important to consider for the future development of moral judgement theories. Extant theories of moral judgement^4–6^ implicitly assume that judgements occur in information-static environments, in which all the necessary information is already available to the decision-maker. To note an exception, Social Intuitionist Model (SIM) specifies how moral judgements change, however only on a longer timescale, and in response to social influence rather than learning new information^6^. Moral judgement theories would benefit from explicitly considering how different morally relevant information is dynamically integrated into judgements. For example, in relation to dual process theory, future research should consider whether consequence-relevant and rule-relevant information is differently integrated in real time. In relation to SIM, it would be interesting to consider whether harm and fairness information is more or less likely to lead to context-specific judgment adjustments than information related to binding foundations (ingroup loyalty, authority, and purity).

### Conclusion

Our findings show that most participants flexibly switch from relying on context-independent norms (generosity, selfishness, and equality), to relying on context-dependent norms (relative generosity, relative selfishness, and indirect reciprocity), as they successfully integrate contextual information. Participants do this in a consistent manner, which suggests that the organizing framework for distributive justice norms has three dimensions reflecting broader virtues: generosity, condemnation of selfishness, and balance. When it comes to judgement of low offers, we present evidence that some participants were particularly careful not to prematurely judge actions as bad without knowing contextual information and adjusted their judgements more strongly when they learned the context. These findings demonstrate that people adjust their moral judgements when they find out new contextual information, switching between different fairness norms that underpin their decisions.

## Methods

### Participants

The sample consisted of 371 University of Melbourne undergraduate students (263 female, 108 male, *M*_*age*_ = 20.57, *SD* = 2.36, range: 18–36 years), who participated in an online study for course credit. The sample size was determined using sensitivity analyses^66^ for two-tailed hypothesis testing (*α* = 0.05) with 80% power^67^ to detect correlation effect sizes larger than *r* = 0.15, accounting for an expectation that a part of the sample (around 10%) will be excluded. The minimum effect size was chosen as to detect small effect sizes, which are common in individual differences research^68^. Fifty eight people were excluded based on predefined data quality criteria: 7 participants failed an attention-check (had given wrong answers in more than 30% of catch trials—see below), 27 participants had responded to over 30% of attention-check questions in the questionnaires incorrectly, 2 participants placed their response in the middle of the scale throughout the task (i.e., did not show any variation in their responding at all, suggesting disengagement with the task), 1 participant did not show any coherence in their responding across identical trials (suggesting random responding), 3 participants drastically changed their context-absent judgement patterns partway through the task (suggesting misunderstanding the task for a substantial period of time), 1 participant consistently rated giving larger amounts as bad and smaller amounts as good (suggesting confusion about scale endpoints, or deliberately providing unexpected behaviour inconsistent with anyone else), and 17 participants misinterpreted the task and judged the Receiver’s as opposed to Dictator’s action (their response correlated positively with the amount that Receiver had given with r > 0.4). Given that we did not preregister the exclusion criteria, we repeated all relevant analyses with the full sample. All results reported below replicate (with slightly weaker, but nevertheless significant coefficients; data not shown), demonstrating that our exclusion criteria did not bias the results. The final sample consisted of 313 participants (222 female, 91 male, *M*_*age*_ = 20.66, *SD* = 2.51, range 18–36 years).

All participants in this study provided a written informed consent via an online form. The study was approved by the Human Research Ethics Committee of the Melbourne School of Psychological Sciences (Ethics ID 1750046), and all methods were performed in accordance with the relevant guidelines and regulations.

### Experimental paradigm

The experiment consisted of three stages. First, participants read a cover story (described below) and a description of the task, and then completed a comprehension test. These instructional materials are available in the supplementary materials (Supplementary Methods S2). Once they passed the comprehension test, they proceeded to complete the experimental task. Finally, they completed a set of questionnaires.

#### Cover story and overview

Participants were presented with a cover story in which they learned about a recently conducted experiment investigating people’s economic decisions. This experiment was entirely fictional, however participants were not informed of this. Participants were informed that their task in the current study would be to morally evaluate these people’s actions in two consecutive steps. In this fictional experiment, a group of people completed a variant of the dictator game consisting of two rounds (see Fig. 1). In the first round, people participating in the experiment were randomly assigned roles of the Dictator and the Receiver, and the Dictator was given $10. The Dictator was termed “the Decision-maker”. The Decision-maker decided on the proportion of the $10 that they would give to the Receiver (*$x*). In the second round, the Round 1 Decision-maker became the Round 2 Receiver and was assigned a new partner, who was then also given $10. The Round 2 Decision-maker decided how much to give to the Receiver (*$y*). Importantly, the Round 2 Decision-maker was able to see how much their partner, the current Receiver, had given in the first round as the previous Decision-maker.

In the current (real) study, participants were instructed to judge only the action of the Decision-makers in Round 2 (i.e. the sharing behaviour of Decision-makers, who themselves had a priori knowledge of the previous sharing behaviours of their respective assigned Receivers). Importantly, they were required to make two consecutive judgements of the same sharing behaviour: For the first judgment, they were only shown how much the Decision-maker shared with the Receiver (*$x*). To give their moral judgement, participants used a continuous scale with endpoints labelled as “bad” and “good”, but no further visual cues. Importantly, participants knew at this stage that the Decision-maker made this decision knowing how much the Receiver had given to their respective partner (*$y*) in the *previous* round playing the same game. Following this initial, context-absent judgement, the amount that the Round 2 Receiver had given in Round 1 (when playing as the Decision-maker) was revealed, and participants were again asked to make the same judgement regarding the Decision-maker’s action as before, again using the continuous scale. Participants judged the same behaviour (i.e. the morality of the current Decision-maker’s sharing behaviour), and the only difference was that participants knew what the Decision-maker “knew” all along: the context in which the Decision-maker’s action occurred.

We piloted this cover story (and the entire experimental protocol) on a separate sample of 18 participants. The piloting session ended with a verbal interview in which the experimenter asked questions concerning believability of the cover story: “Did you have any doubts about the experiment?” and “Was there anything that you found suspicious about the study and the instructions?”. These participants did not report any suspicion that the cover story was not real.

#### Task procedures

Participants were asked to observe a series of independent transactions that various Decision-makers made towards various Receivers, always first without knowledge of the context, and then again after being provided with this context (i.e. the amount $y that the Receiver gave in the previous round, that would have informed the current Decision-maker’s sharing decision). Each trial started with a fixation cross. Next, participants saw a screen displaying how much the current Decision-maker (in Round 2 of the fictional cover study) had given to the current Receiver: “Decision-maker gives: $y”, where y was a number ranging from 0 to 10. The amount that the Decision-maker gave was drawn from a uniform distribution across trials, such that all possible dollar values (no cent amounts) were covered. The response scale with “good” and “bad” labels on each end of the scale was simultaneously presented below the transaction description. Participants indicated their (context-absent) judgement on the scale by clicking at a location between the endpoints using a mouse cursor, and these judgements were mapped onto a numerical scale ranging from − 150 to 150. Next, participants saw the second evaluation screen, providing the context. This screen was identical to the first, but added the crucial information from the previous fictional round: “Receiver gave: $x”, where x was a number ranging from 0 to 10. All possible combinations of x and y values were presented once during the experiment in a randomised order. Participants made a second judgement using the continuous scale, after which the screen was cleared, and a fixation cross marked the start of a new trial.

There were 121 trials in the experiment. Additional attention-check trials were dispersed throughout the task in which participants were required to report the dollar amounts seen in the preceding trial. Participants knew that these trials could occur at random times during the experiment. The attention check trial screen contained the words: “Decision-maker” and “Receiver”, as well as sequences of numbers from ranging from 0 to 10 under each word, referring to the dollar amounts from the previous trial, and participants were instructed to select their answer using their mouse cursor.

Before starting the main task, each participant read the cover story and learned about the task. Participants’ understanding of the task was tested at the end of the instruction session by a comprehensive quiz. The quiz questions specifically tested participants’ knowledge of the rules of the (fictional) two-round dictator game, as well as their comprehension of the two-stage trial structure (for details see the Supplementary Material S2). To ensure full understanding of the task, participants who did not answer all the questions correctly were asked to read the instructions again. Individuals who failed the quiz three times were not invited to complete the experiment.

#### Questionnaires

Various questionnaires were administered after the task procedures. We will analyse and report the questionnaire results in a separate publication, given that this is beyond the scope of the present paper. For completeness, the measures are listed below: We administered the Big Five Inventory 2 (BFI2)^69^, the agreeableness section of the HEXACO Personality Inventory-Revised (HEXACO)^70^, the Questionnaire of Cognitive and Affective Empathy (QCAE)^71^, the Moral Foundations Questionnaire (MFQ), a brief set of self-report measures for political orientation^72^, the Social Dominance Orientation scale (SDO)^73^, and the Global Belief in a Just World Questionnaire (GBJWQ)^74^, and basic demographic measures.

### Analyses of moral judgement patterns

#### Functional principle component analysis

With the goal of characterizing moral judgement patterns across individuals and identifying moral norms (i.e. latent variables giving rise to these patterns of judgements), we used Functional Principle Component Analysis (fPCA)^75,76^. We used the ‘fda’ toolbox v2.2.6^77^ implemented in R v3.5.0. This approach allowed us to fit smooth but flexible spline functions to the patterns of judgements for each participant using a fixed functional form, and to identify dominant modes of variation in the data. There are several benefits of using fPCA: this approach treats the entire spline fit of a participant as a single entity (i.e. a function producing the data), circumventing the problems of data sparsity in high dimensional space present in traditional principle component analyses (PCA) methods^78,79^. The fPCA approach also imposes smoothness constraints and penalties to the data fitting procedure^76,78^. This is necessary because PCA approaches as more commonly used are vulnerable to outliers and may result in principle components that are driven by these outliers, often masking meaningful variability in the data. The constraints and penalties implemented in fPCA minimise the impact of these outliers by accounting for neighbouring data points, and constitute the mathematical implementation of the assumption that true effects of moral norms on judgements across parametrically modulated stimuli will be smooth. This approach has so far been applied in a wide range of fields including medicine, biology, econometrics, and climate studies^80^. In psychology, it has been used to study sources of variation in emotion intensity profiles^81^, and the sources of variation in judgements of tension in music^82^. The current study extends the use of fPCA to judgements across parametrically modulated moral stimuli, with the aim of studying fairness norms latent in moral judgement.

##### Context-absent judgement

To characterise patterns in context-absent judgements across parametrically modulated actions (i.e. sharing different amounts of money), and to identify main components of variability in these patterns, we performed a fPCA. We specified a B-spline basis system with 5 interior knots on points equally spaced over the interval [0, 10] (i.e. the range of values that Decision-maker had given). Each segment in the system was a 4th order polynomial function, resulting in a 9th order basis. The number of knots in the basis system was chosen to be smaller than the number of values that Decision-maker had given in order to impose mild smoothness constraints to the function over the range of values. For each participant, we computed the median judgement across trials (ranging from bad to good) for each value given by the Decision-maker. We fitted the specified spline function system to the data of each participant and performed PCA using these functions as input data. We determined the appropriate number of components by inspection of the scree plot and applying the elbow-criterion (similar to ref.^81^), which means identifying the component solution after which including additional components does not lead to explaining any more significant proportions of the variability. The resulting component solution was rotated using the varimax rotation technique.

##### Context-present judgement

To characterise patterns in context-present judgements across parametrically modulated contextualised actions, and to identify main components of variability in these patterns, we again performed fPCA. To prepare the data for the analysis, we computed median judgements for each magnitude of Decision-maker’s sharing relative to the amount that the Receiver had previously given. The relative sharing of the Decision-maker ranged from − 10 (i.e. Decision-maker gave 0, Receiver gave 10), to 10 (i.e. Decision-maker gave 10, Receiver gave 0). We specified a 9th order B-spline system with 5 interior knots equally spaced over the interval [− 10, 10) (i.e. the relative sharing range). We fitted the defined spline system to the median data (for examples see Fig. 4) and performed fPCA with varimax rotation of the final component solution after applying the same criteria as described above.

##### Judgement adjustment

To characterise patterns in judgement adjustments (i.e. the extent to which people shift their judgement towards “good” or “bad” after learning the contextual information) across parametrically modulated contextualised actions, and to identify main components of variability in these patterns, we again performed fPCA. Similar to analyses of context-present judgements, we specified a 9th order B-spline system with 5 interior knots equally spaced over the interval [− 10, 10]. For each participant we computed judgement adjustment by subtracting the initial context-absent judgement from the final context-present judgement and computed the median judgement adjustment for each magnitude of Decision-makers’ sharing relative to the Receiver (− 10 to 10). We fitted the defined spline system to the median data, and the resulting splines depicted the shift in judgements (ranging from bad to good) for the context-present judgement relative to the context-absent judgement. We performed fPCA with varimax rotation, as described above.

### Quantifying the extent of decision adjustments

Our first aim was to assess whether and to what degree individuals adjusted their judgements. To do so, we derived two simple measures of adjustment for each participant. The first measure was the average absolute adjustment. This measure was calculated as the absolute difference between context-absent and context-present judgements for every trial. This difference was expressed as a percentage of the total scale length, averaged across trials, resulting in the measure of average absolute adjustment for each participant. The second measure was the percentage of trials in which adjustment resulted in a change in judgement valence—i.e., changes in judgements from the left to the right side of the scale, or vice versa. We calculated the means of these two measures within our sample and estimated the corresponding 95% confidence intervals.

### Correlation analyses

The second aim was to examine whether updating is characterised by systematic shifts between pairs of context-independent and context-dependent norms. First, to investigate the relation between context-independent and context-dependent norms, we correlated our measures of importance of specific norms across context-absent and context-present judgements. These analyses captured the relationships between individual differences in context-absent and context-present judgements, treating these two judgements as independent from each-other. As such, these analyses tested whether people who used one norm in context-absent judgements tended to employ another specific norm in context-present judgements. To study individual differences in trial-wise adjustment dynamics, we also calculated the level of adjustment by subtracting the context-absent judgement from the context-present judgement on each trial. As described above, we characterised the individual variation in patterns of context-present adjustment across a continuous range of actions and contexts, using fPCA. To investigate norm shifts across trials—i.e. whether people who used one norm in context-absent judgment tended to adjust their decisions in a particular way in response to contextual information, we correlated the respective principle component scores of context-absent judgements and adjustments.

## Supplementary information

Supplementary Information.

## Data Availability

The datasets generated and analysed during the current study are available in the OSF repository, https://doi.org/10.17605/OSF.IO/XCBUH.

## Code availability

The code used for the analyses presented in this paper are available in the OSF repository, https://doi.org/10.17605/OSF.IO/XCBUH.
