# Supplementary material for: Moral judgements of fairness-related actions are flexibly updated to account for contextual information
Source: Sci Rep. 2020 Oct 20;10:17828. doi: 10.1038/s41598-020-74975-0 (PMC7576593; doi:10.1038/s41598-020-74975-0)
Supplement: Supplementary file 1 — Supplementary Information. [file 41598_2020_74975_MOESM1_ESM.pdf]

Supplementary Materials: Moral Judgements of Fairness-Related Actions are Flexibly Updated to Account for Contextual Information

Milan Andrejević*, Daniel Feuerriegel, William Turner, Simon Laham, Stefan Bode

Melbourne School of Psychological Sciences, The University of Melbourne, Parkville, Victoria 3010, Australia

* Corresponding author; email address: milan.andrejevic@unimelb.edu.au

S1. Correlation Table

	context-absent:		
**context-absent:**	*endorsement of high offers*	*withholding/lenience in judgement of low offers*	*endorsement of even-split offers*
**a)** *endorsement of high offers*	-	-.655***	.332***
withholding/lenience in judgement of low offers	-.655***	-	.018
endorsement of even-split offers	.332***	.018	-
**context-present:**			
**b)** *endorsement of relatively high offers*	.461***	-.419***	.105
lenience in judging relatively low offers	-.295***	.557***	.097
endorsement of similar offers	.068	.108	.47***
**c)** *adjustment to endorse relatively high offers*	- .07	.342***	-.074
lack of adjustment to condemn relatively low offers	.058	-.61***	-.04
adjustment to endorse similar offers	-.246***	.037	.14**

Table S1. Relationships between a) context-absent judgement patterns, b) context-absent and context present judgements, and c) context-absent judgements and adjustments upon presentation of contextual information. Spearman's rank order correlation coefficients are presented here. *** p<.001 ** p<.01

S2. Instruction Materials

Cover Story

The following experiment is designed to investigate how you make decisions regarding other people’s behaviour. Today’s session should not take more than 1.5 hour altogether.

This experiment is part of a larger project studying people's economic decisions. Before you start the task it is important to learn about the first experiment we conducted as a part of this project. Please read all instructions carefully and make sure you understand everything because otherwise you will not do today's task correctly. The instruction session has **a test** at the end. **This is to ensure that you have understood what you need to do in the task. You will have to pass this test to be able to do today's task**.

Background

In the first experiment of the project 300 participants completed a task consisting of two rounds:

[Two sets of animated slides were embedded here explaining Round 1 and Round 2 respectively. To illustrate how the slides looked like we have stripped animations away and are pasting static images of these slides here.]

[SLIDE SET 1:]

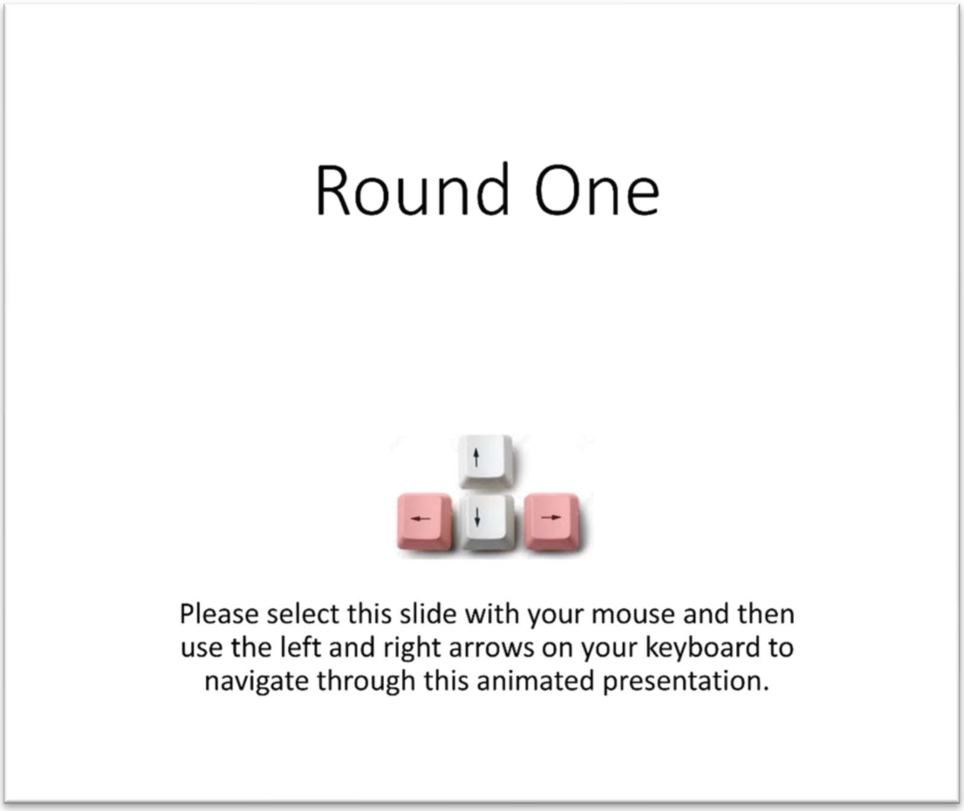

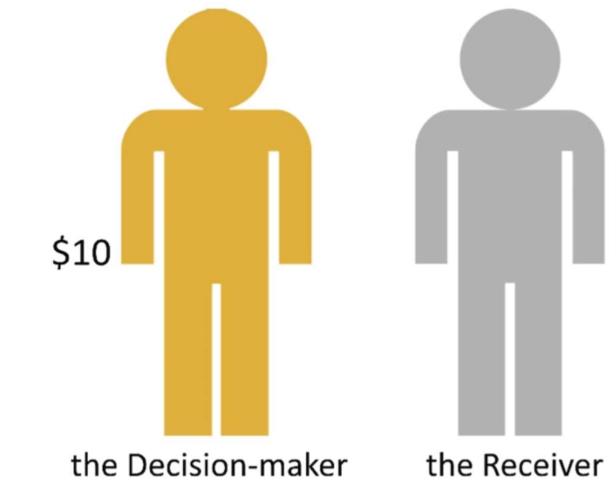

First, people were grouped into pairs of two, and arbitrarily assigned the roles of a “Decision-maker” and a “Receiver”.
The Decision-maker was given 10 dollars.

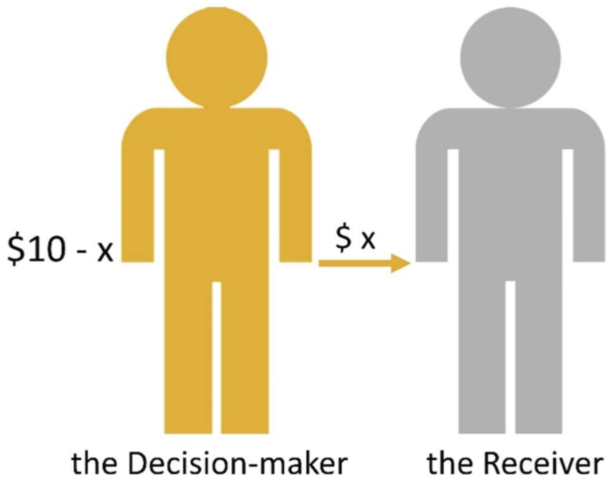

The Decision-maker decided how much of the $10 to share with the Receiver. They could decide to give any amount ranging between 0 and 10 dollars.
The Decision-maker gave *x* dollars to the Receiver.

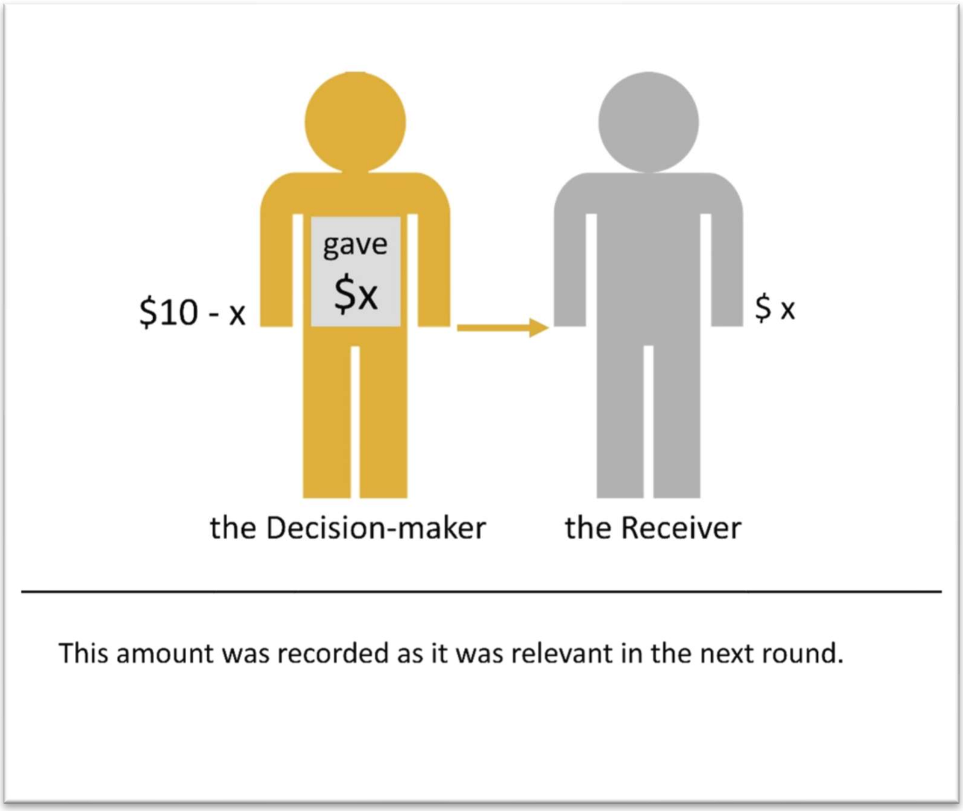
gave
$x
$10 - x
$ x
the Decision-maker
the Receiver
This amount was recorded as it was relevant in the next round.

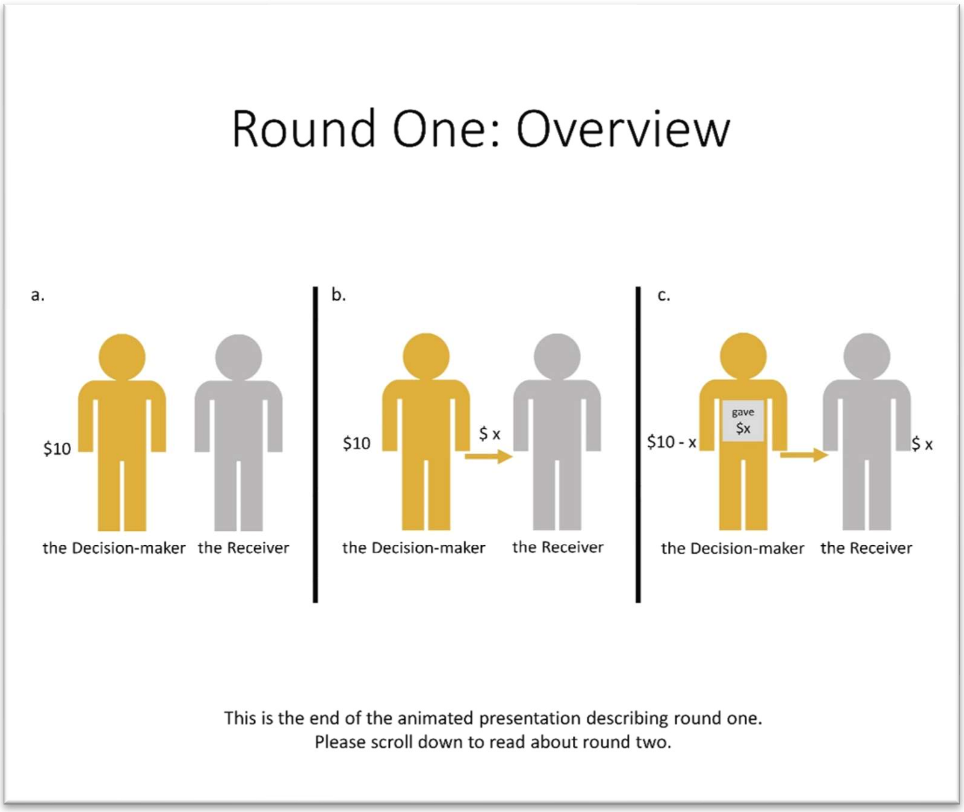
Round One: Overview
a.
$10
the Decision-maker
the Receiver
b.
$10
$ x
the Decision-maker
the Receiver
c.
gave
$x
$10 - x
$ x
the Decision-maker
the Receiver
This is the end of the animated presentation describing round one.
Please scroll down to read about round two.

[SLIDE SET 2:]

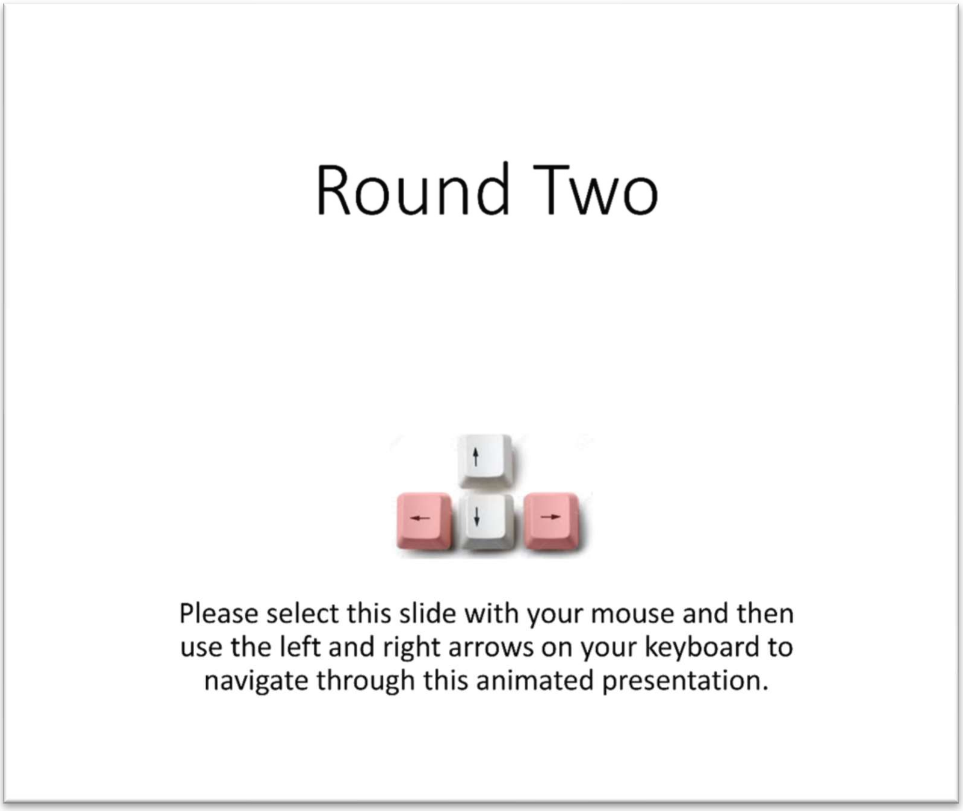
Round Two
Please select this slide with your mouse and then use the left and right arrows on your keyboard to navigate through this animated presentation.

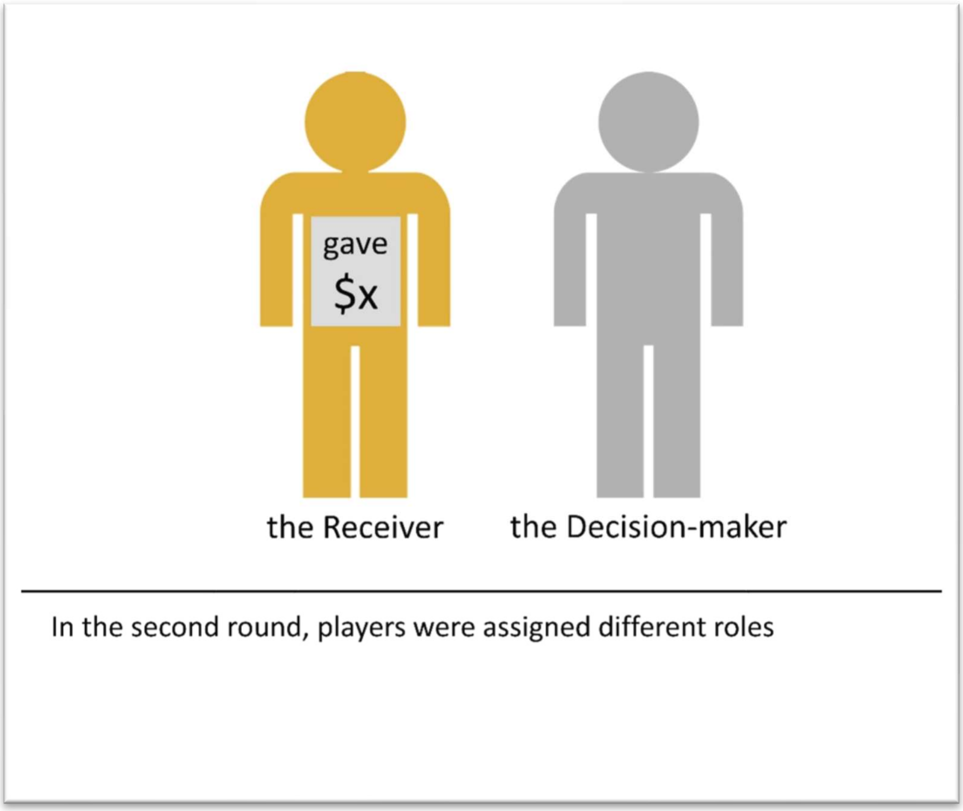
gave
$x
the Receiver
the Decision-maker
In the second round, players were assigned different roles

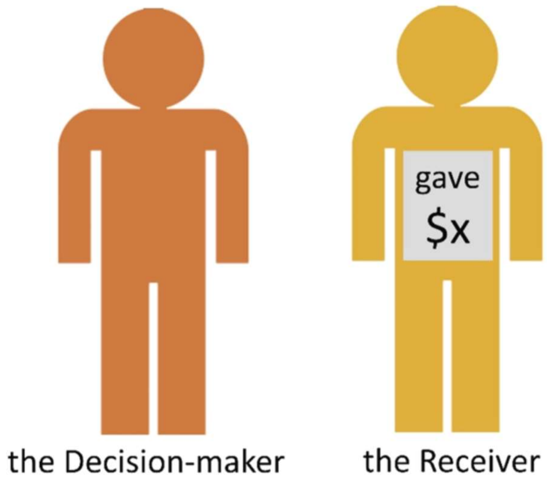

In the second round, players were assigned different roles and new partners.
In our example, the yellow person who was the Decision-maker in the first round, became the new Receiver. The new Receiver was paired with a new partner who became the Decision-maker.

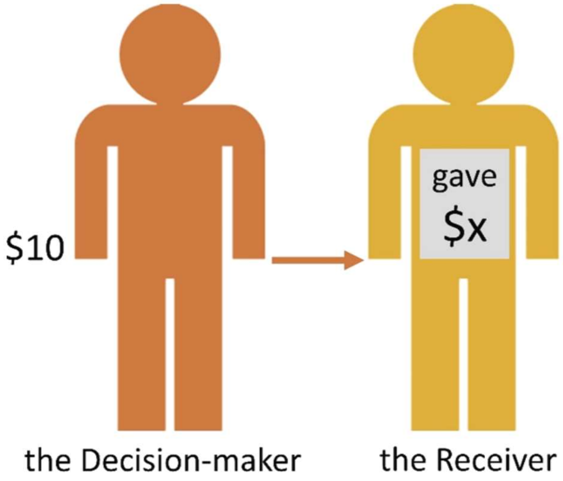

The new Decision-maker was given 10 dollars and decided how much to give to the Receiver.
Importantly, in the second round, the **new Decision-maker was able to see** how much their partner, the current Receiver, had given in the first round.

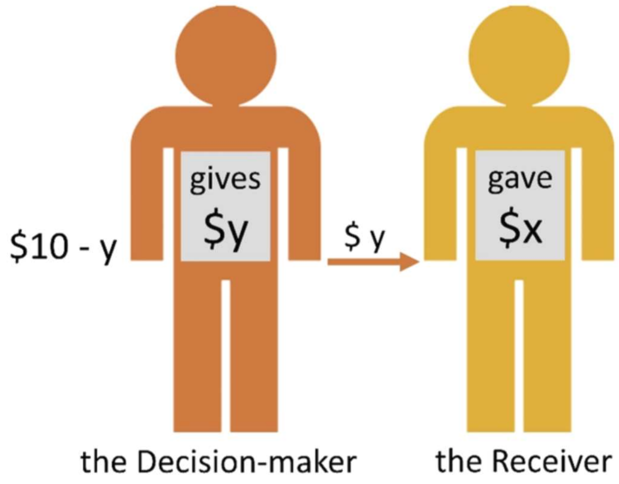

The Decision-maker gave *y* dollars to the Receiver.
The amount that the Decision-maker gave was also recorded as it is relevant for today's experiment.

Round Two: Overview

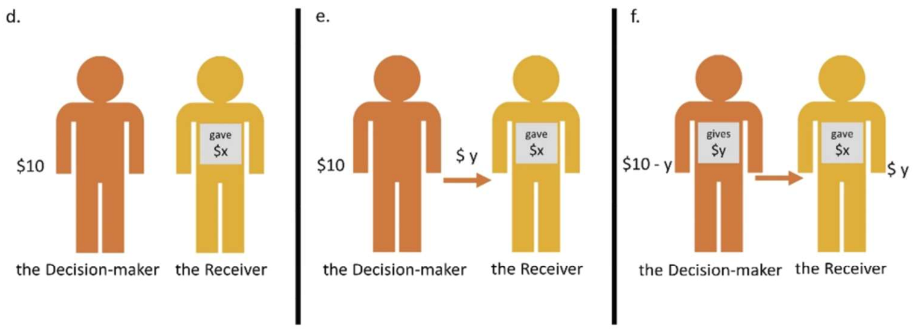

This is the end of the animated presentation describing round two.
Please scroll down to continue reading instructions.

A large number of people completed these two rounds. Participants were aware that the decisions that they made in either round had real economic consequences. They were deciding how much money they and their partner would receive at the end of the experiment.

Your task today is to observe what happened **in the second round** of the experiment. You will be shown how much the Decision-maker gave, with the Decision-maker knowing how much the Receiver had given in the first round. Before we move onto describing today's task in detail, please make sure you understand the experiment, and reread the instructions above until everything is clear.

Today's Task

The purpose of today's study is to investigate what you, an impartial observer, think about how people behaved in the experiment described above.

You will watch some of the decisions that our participants made as they played the role of a Decision-maker ***in the second round***. **You will see the amount the Decision-maker decided to give to the Receiver, knowing how much the Receiver had given in the previous round.** Your task is to evaluate whether their action (i.e. their decision to give Y dollars) was morally good or bad. You will observe multiple pairs of participants, with one pair in each trial.

Here is a description of what will happen in each trial:

[SLIDE SET 3:]

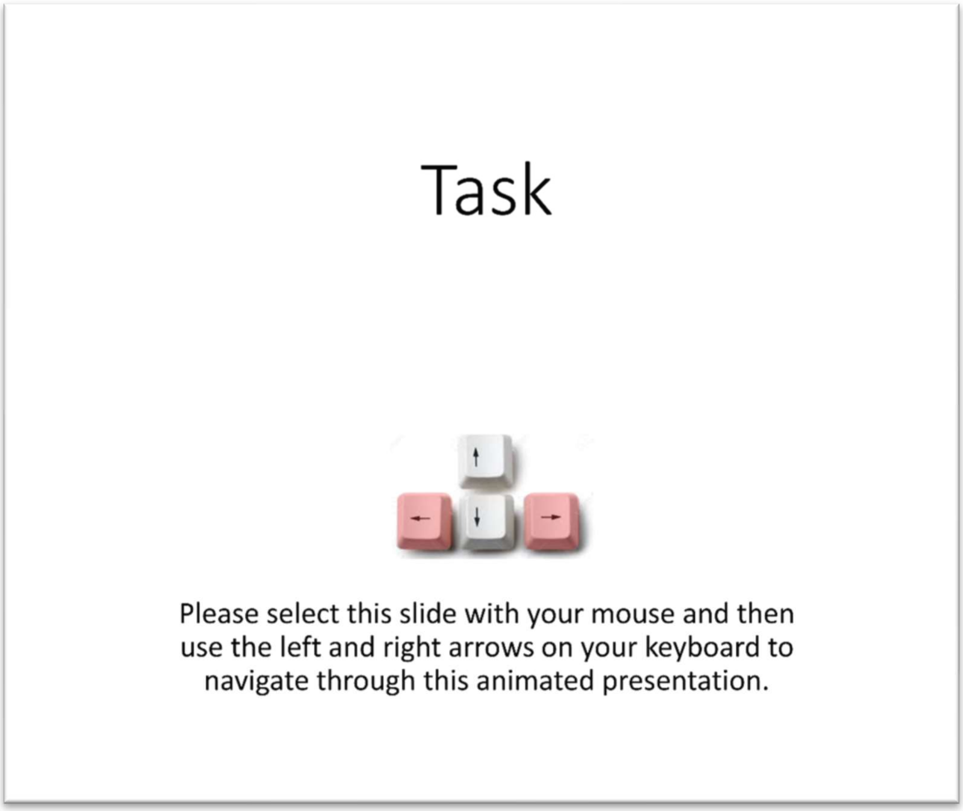

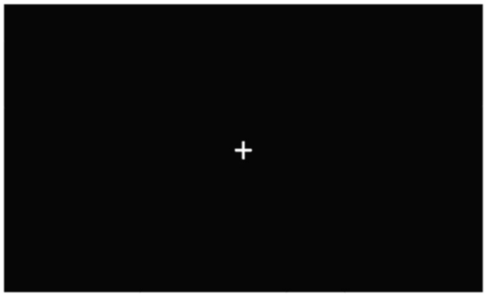

1 – 2 s

Each trial will start with a cross presented in the middle of the screen - please focus on this point on the screen.

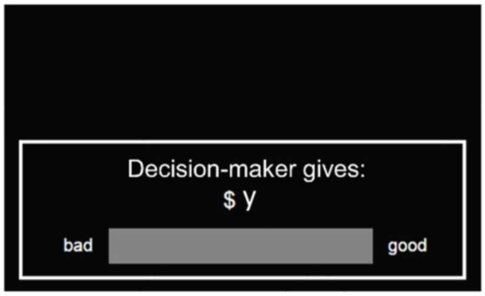

5 s maximum

Next you will see the text: "Decision-maker gives: $Y". Y will be a number showing how many dollars were given to the Receiver.
A response bar will appear underneath the text for you to make a moral judgement.

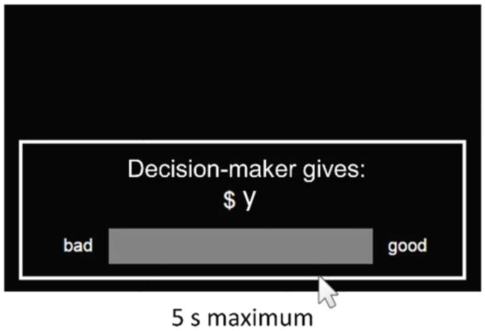

5 s maximum

Use your mouse cursor to left click at a position on a response bar, depending on how morally good or bad you believe the Decision-maker's action was.
You have a maximum of 5 seconds to respond.

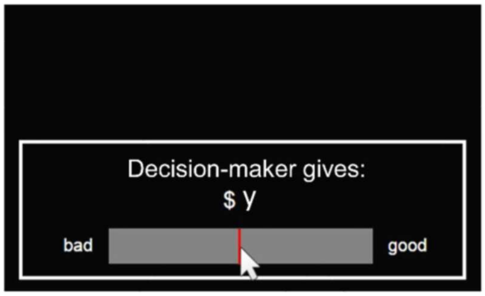

5 s maximum

Please make your moral judgement about the action of the Decision-maker. In this task you are not judging the person, but are judging their action.
It is essential that you respond as quickly and accurately as possible and that you **always** give a response.

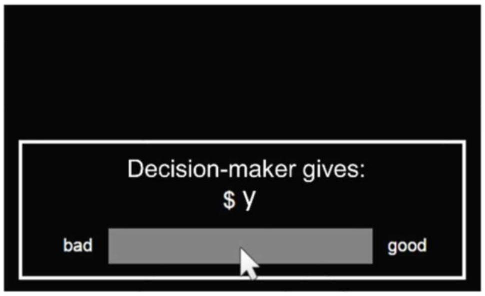

5 s maximum

Next you will see how much the current Receiver had given in the first round of the experiment.
The current Decision-maker whose action you just judged, knew this at the time they made their decision.

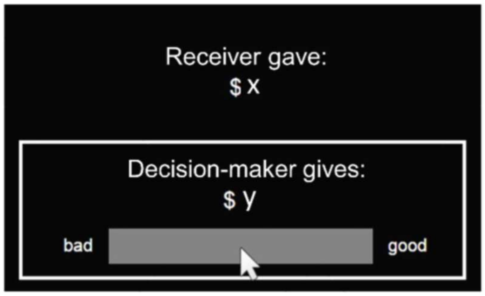

5 s maximum

After knowing how much the Receiver had previously given, you must now **make another moral judgement about the action of the current Decision-maker.**
To clarify, you need to judge the same action you judged the first time.
Do not make your judgement about the action of the current Receiver**.**

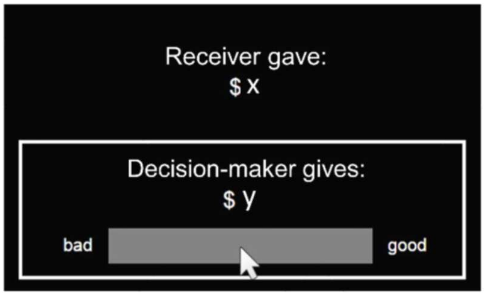

5 s maximum

You must again left click at a position on the response bar, depending on how morally good or bad you believe the Decision-maker's action was.
The trial will end when you make a response, or after 5 seconds.

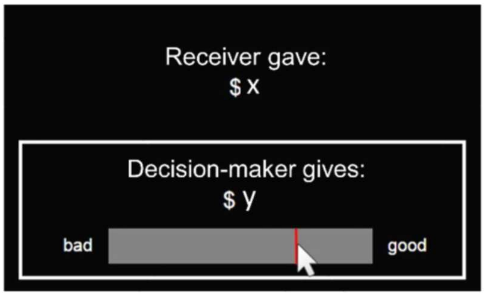

5 s maximum

It is essential that you **respond as quickly and accurately as you can, and always give a response**.

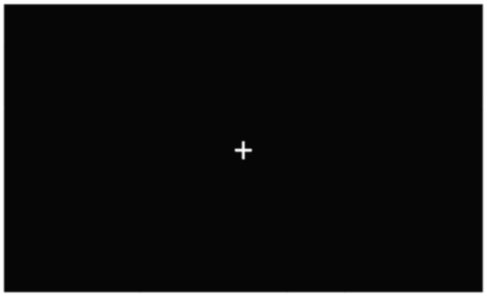

1 – 2 s

Once you have selected your answer, you will see a white cross in the middle of the screen, and a new trial will begin.

Task: Overview

a.

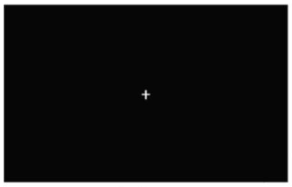

1 – 2 s

b.

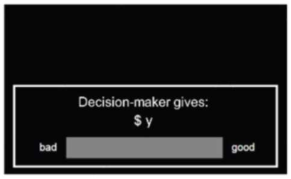

5 s maximum

Please judge the action of the Decision-maker.

c.

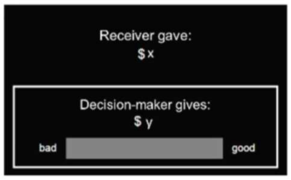

5 s maximum

Please judge the action of the same Decision-maker again.

This is the end of the animated presentation describing one task trial.
Please scroll down to read more about the task.

This task requires your full attention - please try to respond as quickly and accurately as possible. If you do not make a response in either of the two steps, you will receive a message asking you to respond faster.

After some trials you will see an extra **attention-check task screen**. You will be asked to report the dollar amounts that the Decision-maker and Receiver had given in that trial. You will see the following response screen:

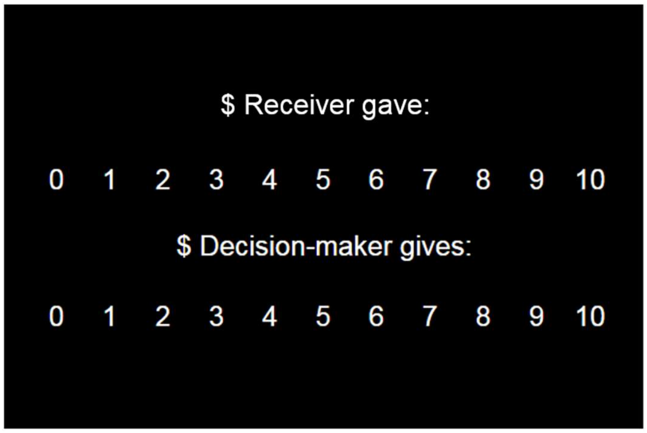

Your task here is to **left click on the dollar amounts you saw in the trial**.
Precision is not essential here - if your response is 1 dollar off, we will still count it as correct. It is just essential that you are paying attention during the task. This response screen could appear **after any trial** during the experiment, so it is essential that you pay attention during all trials. If you respond incorrectly on too many trials the entire experiment will fail.

Before we begin the task, we will test your comprehension of the instructions. Please make sure you understand the task and reread the instructions above until everything is clear. All your answers need to be correct for you to be able to participate in this experiment.

Comprehension Test

To participate in the study you will need to **answer all questions in this test correctly**. Please read each item carefully, and if unsure of the correct answer, you are welcome to use the "back" button on this page to go to the instructions and read again. Your answers will not be lost if you go back and forth. Only press the submit button once you are sure that all answers are correct.

Understanding what happened in the economic decision experiment is essential for today's task. Which of the following is true about the experiment:

	True	False
The roles of the Decision-maker and the Receiver were assigned at the start of Round 1 and stayed the same for Round 2.	○	○
Decision-makers from Round 1 were assigned Receiver roles in Round 2.	○	○
In Round 2, the Receiver decided whether or not to accept the Decision-maker's offer.	○	○
In Round 2, the pair of participants from Round 1 swapped roles and played again with each other.	○	○
In Round 2, the Receiver was aware how much the Decision-maker received in Round 1 when they played the role of the Receiver.	○	○

In Round 2, the Decision-maker was aware how much the Receiver gave in Round 1 when they played the role of the Decision-maker.	○	○

Your task today will be to observe how participants from our previous experiment behaved. Which of the following statements about today's task is true:

	True	False
Your task is to evaluate actions of the Decision-maker in Round 1 using a response bar.	○	○
In each trial of the task, you will judge the Round 2 Decision-maker's action two times.	○	○

As a first step in each trial you will see the following screen:

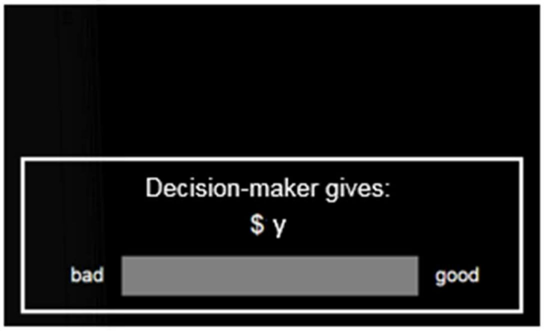

Which of the following statements about this screen is true:

	True	False
The Decision-maker has decided to give $y to you.	○	◉
Instead of '$y' you will see an actual number on the screen.	◉	○
You have a maximum of 5 seconds to make a response.	◉	○
Your task is to evaluate the Decision-maker's action.	◉	○
You will respond with your mouse.	◉	○
You should respond on every trial as quickly and accurately as possible.	◉	○

As a second step in each trial you will see the following new information on the screen:

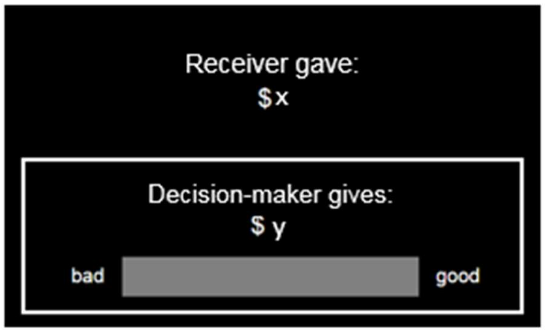

Which of the following statements about this step are true:

	True	False
The current Receiver had given the current Decision-maker x dollars in Round 1.	○	◉
The current Receiver had given x dollars to another person in Round 1.	◉	○
You have a maximum of 5 seconds to make a response.	◉	○
Your task is to evaluate the Receiver's action.	○	◉
Your task is to evaluate the Decision-maker's action.	◉	○

To make sure you are paying attention the task includes the attention-check task screen. Which of the following is true about this attention-check task:

	True	False
In this task you will rate on a scale from 1 to 10 what you think about what the Decision-maker and the Receiver did in the last trial.	○	◉
In this task you will report the dollar amounts you saw in the last trial.	◉	○
This attention-check task will appear after every trial.	○	◉
